# Expression of epithelial-mesenchymal transition-related genes increases with copy number in multiple cancer types

**DOI:** 10.18632/oncotarget.8371

**Published:** 2016-03-25

**Authors:** Min Zhao, Yining Liu, Hong Qu

**Affiliations:** ^1^ School of Engineering, Faculty of Science, Health, Education and Engineering, University of the Sunshine Coast, Maroochydore, Queensland, 4558, Australia; ^2^ Center for Bioinformatics, State Key Laboratory of Protein and Plant Gene Research, College of Life Sciences, Peking University, Beijing, 100871, P.R. China

**Keywords:** pan-cancer, copy number variation, cancer genomics, epithelial-mesenchymal transition (EMT), gene expression

## Abstract

Epithelial-mesenchymal transition (EMT) is a cellular process through which epithelial cells transform into mesenchymal cells. EMT-implicated genes initiate and promote cancer metastasis because mesenchymal cells have greater invasive and migration capacities than epithelial cells. In this pan-cancer analysis, we explored the relationship between gene expression changes and copy number variations (CNVs) for EMT-implicated genes. Based on curated 377 EMT-implicated genes from the literature, we identified 212 EMT-implicated genes associated with more frequent copy number gains (CNGs) than copy number losses (CNLs) using data from The Cancer Genome Atlas (TCGA). Then by correlating these CNV data with TCGA gene expression data, we identified 71 EMT-implicated genes with concordant CNGs and gene up-regulation in 20 or more tumor samples. Of those, 14 exhibited such concordance in over 110 tumor samples. These 14 genes were predominantly apoptosis regulators, which may implies that apoptosis is critical during EMT. Moreover, the 71 genes with concordant CNG and up-regulation were largely involved in cellular functions such as phosphorylation cascade signaling. This is the first observation of concordance between CNG and up-regulation of specific genes in hundreds of samples, which may indicate that somatic CNGs activate gene expression by increasing the gene dosage.

## INTRODUCTION

Epithelial-mesenchymal transition (EMT) is the transformation of an epithelial cell into a mesenchymal cell, the latter of which is a critical cell type for the initiation of cancer metastasis [1–3]. During this morphological and cellular change, cells also acquire different cellular functions. While epithelial cells have the tight intercellular connections due to their cellular junctions, mesenchymal cells have only loose connections at focal points. In addition, epithelial cells that transform into mesenchymal cells through EMT lose their cellular polarity. Mesenchymal cells are migratory and invasive, and resist to cell senescence, inflammation, immunotherapy and chemotherapy [2, 4]. Therefore, understanding EMT may facilitate the development of diagnostic biomarkers for cancer diagnosis.

To explore the molecular mechanism of EMT at a systems-biology level, we developed a literature-based gene resource specific for EMT. In total, 377 human EMT-implicated genes were collected from thousands of published articles, primarily single nucleotide mutation-based studies. Reports of systematic data mining for larger genomic variations related to EMT, such as gene copy number variations (CNVs), are still lacking. As a genomic mutational event, CNV often results in a variable number of repeated DNA fragments with lengths ranging from kilobases to megabases, even encompassing entire genes [5]. Using array-based high-throughput technology, large-scale CNVs were detected in The Cancer Genome Atlas (TCGA) projects [6, 7]. CNVs can be classified into two major groups: copy number gain (CNG, an increased number of copies of a gene in the genome) and copy number loss (CNL, a reduced number of copies of a gene in the human genome). By altering the gene dosage (the exact number of copies of a particular gene in a genome [8]) from its normal diploid state, tthese deleted or duplicated DNA fragments often profoundly affect gene expression [8].

CNV is known to correlate with altered gene expression in different cancers [9]. While such studies of single cancer types have been useful for gene discovery, they have not validated CNVs across different cancer types. To comprehensively evaluate the relationship between CNVs and EMT-implicated gene expression changes, we performed a pan-cancer CNV analysis.

## RESULTS

### The EMT-implicated genes with frequent CNGs highlighted the role of proteoglycans in cancer metastasis

To explore somatic CNV information at the pan-cancer level, we first downloaded 377 human EMT-implicated genes from our literature-based database, dbEMT [10]. Then, we aligned the genomic coordinates of these 377 EMT-implicated genes with CNVs of major cancers from the TCGA pan-cancer study [11]. In total, 365 genes overlapped with cancer CNVs in certain genomic regions. In order to focus on CNVs with precise gain or loss information, we removed non-informative CNVs that lacked control tissue (see Materials and Methods). As EMT can promote cancer metastasis, we assumed that CNGs of EMT-implicated genes would be the driving force for gene expression changes related to EMT. We counted the number of TCGA cancer samples with CNGs or CNLs and established a cut-off so that we could extract the most informative CNG events. Our criterion was that the number of samples with CNGs in an EMT-implicated gene must be at least twice the number of samples with CNLs. In this way, we harvested a total of 212 genes for further functional annotation and integrative gene expression analysis (Table S1).

**Figure 1:**
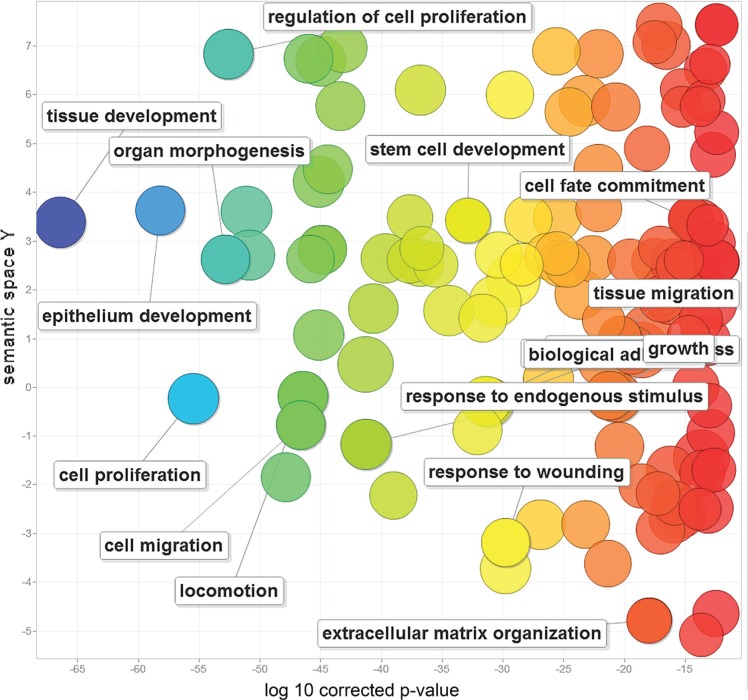
Gene ontology analysis of 212 human EMT-implicated genes with frequent CNGs The scatterplot shows the gene ontology (GO) clusters for the 212 EMT-implicated genes in a two-dimensional space derived through application of multidimensional scaling to a matrix of the semantic similarities of the GO terms. Bubble colors indicate the frequency of a GO term in the GOA database (bubbles with more general terms are red), while bubble sizes indicate the log of the corrected *P*-value (bubbles with smaller corrected *P*-values are larger).

We first performed a gene ontology analysis to determine the functions of the 212 EMT-implicated genes with frequent CNGs. Not surprisingly, the majority of the functions were related to cell migration (corrected *P*-value = 2.09E-46), cell proliferation (corrected *P*-value = 2.99E-55), and cell fate commitment (corrected *P*-value = 2.54E-14) (Figure 1). These EMT-implicated genes may also participate in epithelium development (corrected *P*-value = 5.699E-58), stem cell development (corrected *P*-value = 1.218E-32), and extracellular matrix organization (corrected *P*-value = 9.741E-18). Intriguingly, these 212 genes are also involved in the responses to wounding (corrected *P*-value = 1.542E-29) and endogenous stimuli (corrected *P*-value = 4.951E-41).

Further biological pathway analysis of the 212 EMT-implicated genes revealed 425 significantly enriched biological pathways (all corrected *P*-values less than 0.01, and with five or more annotated genes) (Table S2). The most significant pathway was “proteoglycans in cancer,” with which 39 genes were associated (Table 1, corrected *P*-value = 5.78E-25). Cell surface proteoglycans act as either inhibitors or promoters of cancer metastasis, depending on the type of cancer [12]. For instance, the proteoglycan syndecan-1 is highly expressed on the membranes of epithelial cells and can promote cell adhesion, a key step in EMT [12].

**Table 1:** The top 10 pathways enriched for the 212 EMT-implicated genes with frequent CNGs

Pathway	*Q*-value^1^	#G^2^	EMT List
Proteoglycans in cancer	5.78E-25	39	MYC,HGF,ERBB2,ITGA5,ITGB1,ITGB3,PTK2,STAT3,DDX5,TWIST1,MET,BRAF,PIK3CA,WNT3A,PLAUR,FGFR1,SDC1,RAC1,VEGFA,ROCK2,KRAS,RAF1,VTN,HBEGF,WNT1,CAV1,TGFB1,TGFB2,MAPK14,IGF1R,EGFR,CD44,PAK1, MIR21,PRKCA,ROCK1,TNF,MAPK1,MAPK3
Pathways in cancer	4.93E-21	41	MYC,CDKN1B,HGF,ERBB2,ITGA6,ITGB1,PTGS2,PTK2,STAT3,STAT5A,AXIN1,STAT5B,AXIN2,MET,BMP2,BMP4,BRAF,PIK3CA,WNT3A,FGFR1,FGFR2,KIT,RAC1,VEGFA, KRAS,RAF1,WNT1,SHH,LAMA5,BIRC2,PPARG,GSK3B,TGFA,TGFB1,TGFB2,IGF1R,EGFR,PRKCA,MAPK1,MAPK3,EPAS1
Integrated pancreatic cancer pathway	4.93E-21	29	MYC,CDKN1B,SP1,ERBB2,EZH2,PTGS2,STAT5A,PIK3CA, FGFR1,RAC1,VEGFA,KRAS,RAF1,ANXA1,WT1,SHH,GSK3A,TGFB1,LEFTY1,MAPK14,IGFBP3,EGFR,EGR1,PAK1, PRKCA,TNF,MAPK1,MAPK3,MAPK7
MicroRNAs in cancer	4.56E-16	34	MYC,CDKN1B,ERBB2,ITGA5,EZH2,ITGB3,PTGS2,STAT3,BMI1,MET,PIK3CA,WNT3A,NOTCH2,VEGFA,KRAS,RAF1,VIM,ZEB1,TGFB2,MIR137,MIR15B,EGFR,TP63,MIR194–1,CD44,MIR21,PRKCA,PRKCE,ROCK1,MIR200C,MAPK1,MAPK7,FSCN1,MIR23A
Focal adhesion	5.94E-16	29	HGF,ERBB2,ITGA6,ITGA5,ITGB1,ITGB3,ITGB4,ZYX,PTK2,MET,BRAF,PIK3CA,RAC1,VEGFA,ROCK2,RAF1,FLT1,VTN,LAMA5,BIRC2,CAV1,GSK3B,IGF1R,EGFR,PAK1,PRKCA,ROCK1,MAPK1,MAPK3
MicroRNAs in cardiomyocyte hypertrophy	3.98E-14	21	STAT3,PIK3CA,WNT3A,FGFR2,RAC1,ROCK2,RAF1,EDN1,GSK3B,TGFB1,MAPK14,IGF1R,MIR15B,LRP6,MIR21,ROCK1,TNF,MAPK1,MAPK3,MAPK7,MIR23A
Prolactin signaling pathway	1.58E-12	17	MYC,ERBB2,ITGB1,PTK2,STAT3,GAB2,STAT5A,STAT5B, PIK3CA,RAC1,RAF1,YWHAZ,GSK3B,MAPK14,PAK1,MAPK1,MAPK3
ErbB signaling pathway	1.58E-12	18	MYC,CDKN1B,ERBB2,PTK2,STAT5A,STAT5B,BRAF,PIK3CA,KRAS,RAF1,HBEGF,GSK3B,TGFA,EGFR,PAK1,PRKCA,MAPK1,MAPK3
IL-3 signaling Pathway	2.26E-12	19	PTK2,STAT3,GAB2,STAT5A,STAT5B,PIK3CA,HSPB1,RAC1,KRAS,RAF1,YWHAZ,GSK3A,GSK3B,MAPK14,PAK1,PRKCA,MAPK1,MAPK3,MAPK7

1 *Q*-values: the raw *P*-values of the hypergeometric test were corrected by Benjamini-Hochberg multiple testing correction.

2 *G*: the number of EMT-implicated genes associated with the pathway.

We also identified 17 genes belonging to the “prolactin signaling pathway” (corrected *P*-value = 1.58E-12). The prolactin receptor is widely expressed in extrapituitary cells of breast, liver, pancreas, and gastrointestinal tissues [13]. Prolactin can transport water and electrolytes through the mucosal membrane [13]. More interestingly, the proteoglycans and the prolactin signalling pathways are not isolated from each other [14]. For instance, acidic glycosaminoglycans (proteoglycans) have been detected in prolactin secretory granules [14]. Taking these data together, frequent CNGs appear to occur for genes associated with proteoglycans and the prolactin signaling pathway in EMT.

### The genes with concordance between CNG and up-regulation include numerous regulators of apoptosis

**Figure 2:**
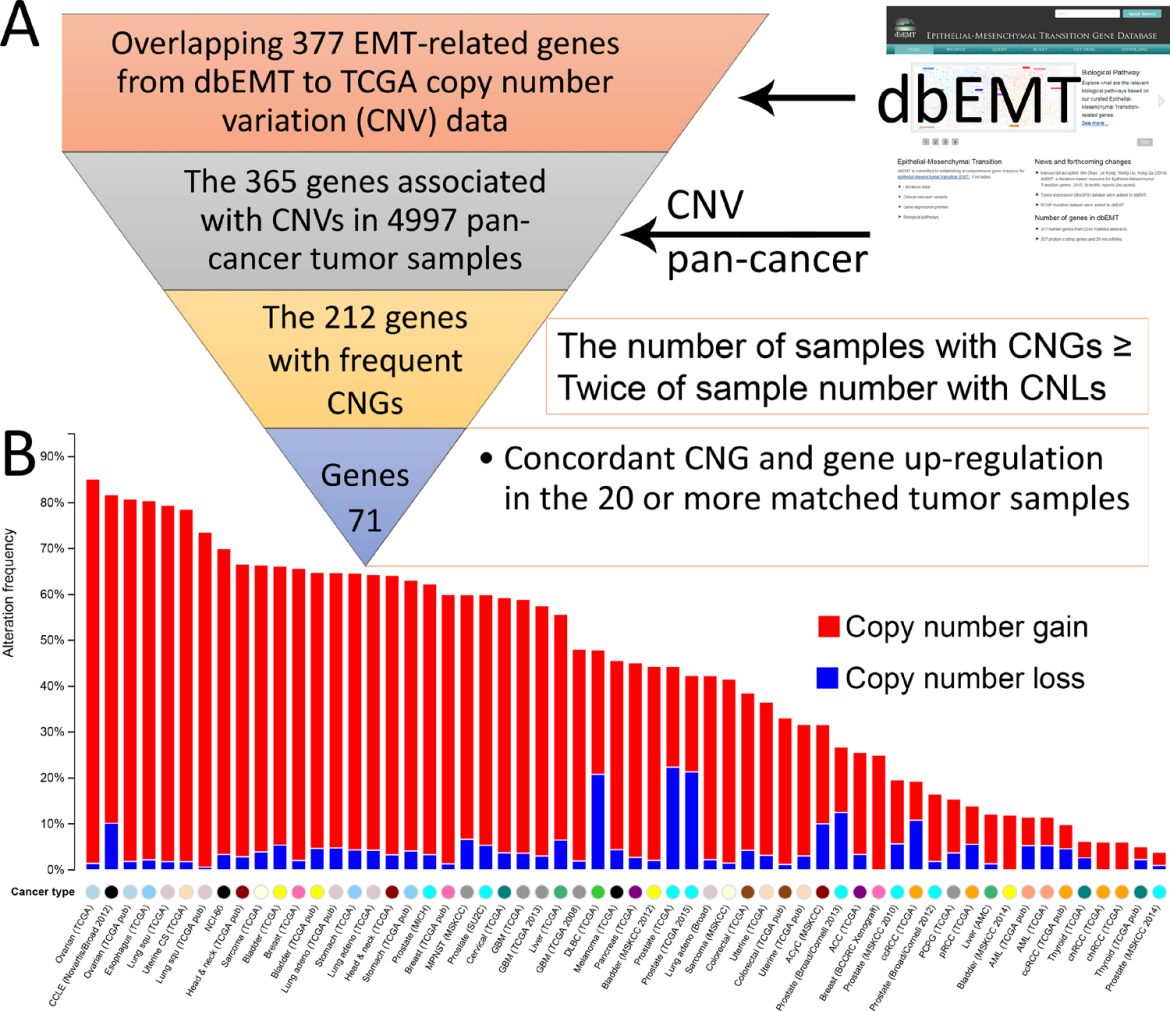
Collection of 71 EMT-implicated genes with increased gene expression induced by CNGs (**A**) The computational pipeline for identifying 71 EMT-implicated genes with concordance between CNG and up-regulation; (**B**) the global CNV patterns across multiple cancers for 71 EMT-implicated genes with increased gene expression induced by CNGs.

We then evaluated whether CNGs in the TCGA samples correlated with the up-regulation of the same EMT-implicated genes (Figure 2). To determine whether the expression of these EMT-implicated genes was higher in tumor samples than in normal samples, we calculated Z-scores to characterize the over-expressed values in a specific TCGA sample. We established a cut-off value of 2 for the calculated Z-score to identify highly expressed EMT-implicated genes in specific TCGA samples.

**Figure 3:**
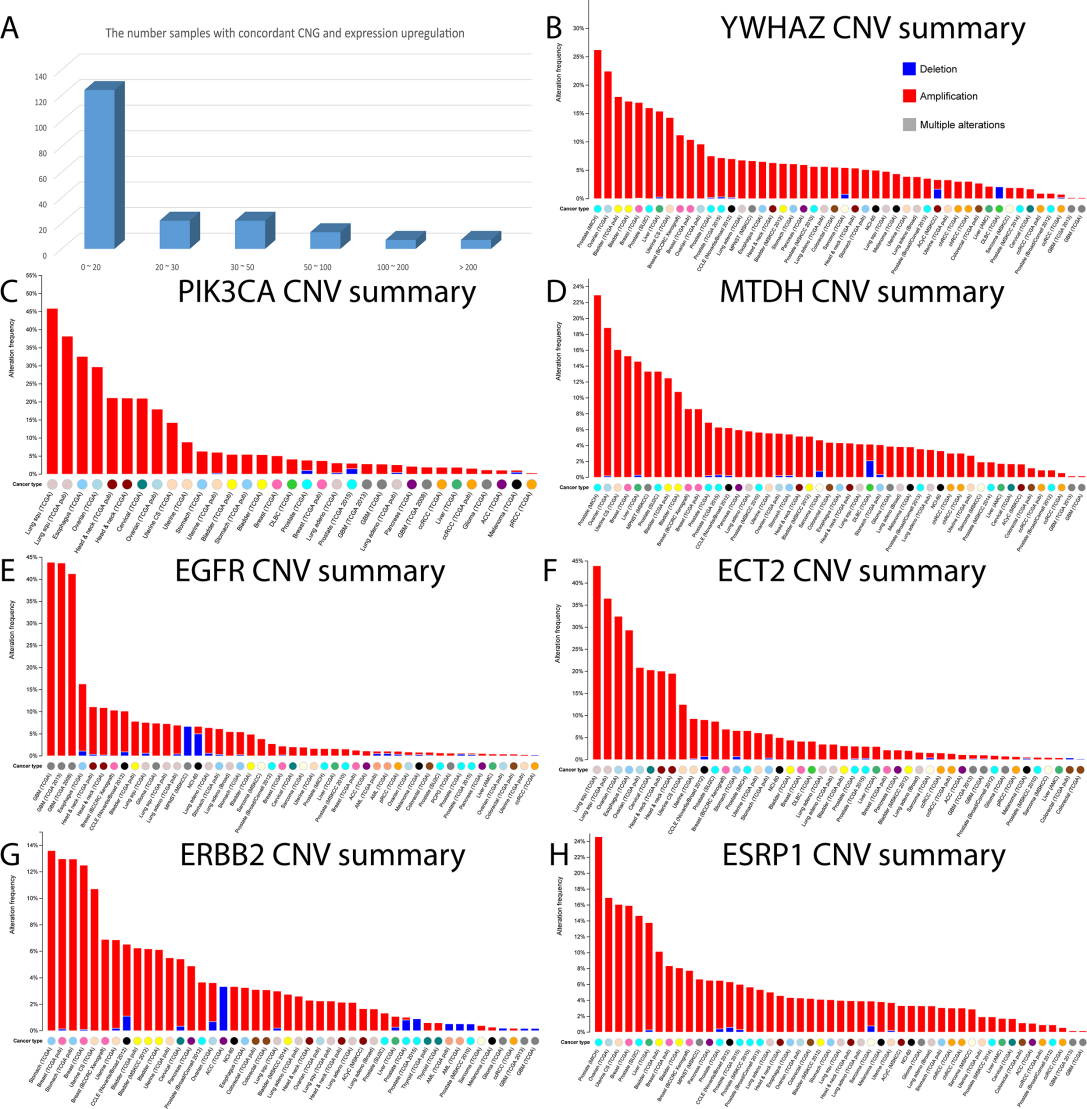
The number of genes with concordance between CNG and up-regulation, and the global CNV mutational pattern (**A**) The number of EMT-implicated genes with concordance between CNGs and up-regulation in different tumor samples. (B-H) The CNV landscape in multiple cancer datasets in the cBio portal for: (**B**) *YWHAZ*, (**C**) *PIK3CA*, (**D**) *MTDH*, (**E**) *EGFR*, (**F**) *ECT2*, (**G**) *ERBB2*, and (**H**) *ESRP1*.

By focusing on the matched TCGA tumor samples with both expression and CNV information, we unveiled 195 EMT-implicated genes with increased gene expression and a concordant gain of gene copy number. To further confirm the potential CNG-induced gene expression change, we counted the number of samples with concordance between up-regulation and CNG for each of the 195 EMT-implicated genes (Figure 3A). The majority of the genes (61%) exhibited concordance in fewer than 20 samples. Based on the criterion of concordant CNG and gene up-regulation in 20 or more matched tumour samples from various cancers, we compiled a list of 71 EMT-implicated genes with high confidence (Table S3). Among these 71 genes, 41 were positive regulators in response to stimuli (corrected *P*-value = 1.913E-22) (Table S4). Many of these genes were also involved in basic cellular processes such as apoptosis (42 associated genes, corrected *P*-value = 1.42E-21) and the MAPK cascade (31 associated genes, corrected *P*-value = 6.96E-16). In summary, our step-by-step filtering approach identified a subset of EMT-implicated genes with frequent CNGs and concordant gene up-regulation that are involved in basic cellular processes.

To visualize the frequently mutated genes that are common to multiple cancers, we overlapped the 71 EMT-implicated genes with pan-cancer CNV data (Figure 2B). In the TCGA ovarian serous cystadenocarcinoma cohort, there were 493 cases (85.10%) with an altered copy number of at least one of these genes (Table S5). Interestingly, these 71 genes were also mutated in 813 cell lines (81.70%) in the Cancer Cell Line Encyclopedia (CCLE) dataset. As the CCLE is comprehensive with respect to cancer types, the high mutation rates observed in the 71 genes reflect their recurrent mutational patterns. Over 80% of the esophageal carcinoma patients had at least one deletion event in one of the 71 EMT-implicated genes. A similar prevalence of CNVs (> 60% cases) was found in 11 other cancer types (bladder urothelial carcinoma, breast invasive carcinoma, esophageal carcinoma, head and neck squamous cell carcinoma, lung adenocarcinoma, lung squamous cell carcinoma, malignant peripheral nerve sheath tumor, metastatic prostate cancer, sarcoma, stomach adenocarcinoma, and uterine carcinosarcoma) and in NCI-60 cell lines. In addition, in these 12 cancer types and two cell line datasets, CNGs were overwhelmingly more prevalent than CNLs (Figure 2B). The consistent detection of these highly frequent mutations in multiple cancers and cell lines may imply that frequent CNGs in these 71 EMT-implicated genes are important for cancer progression.

Intriguingly, we found seven genes with concordance in over 200 tumour samples, including *YWHAZ* (345 samples), *PIK3CA* (313), *MTDH* (293), *EGFR* (285), *ECT2* (271), *ERBB2* (220), and *ESRP1* (219) (Figure 3B–3H). An additional seven genes exhibited concordance in over 110 samples: *SCRIB* (157), *NDRG1* (151), *EIF5A2* (150), *ZNF217* (145), *KRAS* (138), *MYC* (135), and *TP63* (113). The high frequency of concordance for these genes indicates that the CNGs may drive the increases in gene expression. In addition, 10 of the 14 identified genes were key “regulators of apoptosis” (GO:0042981, corrected *P*-value = 2.35E-5). *YWHAZ* belongs to the 14–3-3 gene family, which can bind to phosphoserine-containing proteins, and participates in the PI3K-Akt signaling pathway in certain cancers [15, 16]. CNGs in this gene were presented in over 25% of the cases from a lethal castration-resistant prostate cancer cohort from Michigan (Figure 3B). In this same prostate cancer cohort, there were also frequent CNGs of *MTDH* and *ESRP1* (Figure 3D, 3H). There were repeat copies of *PIK3CA* and *ECT2* in approximately 45% of patients in a TCGA lung squamous cell carcinoma cohort (Figure 3C, 3F). Likewise, there were frequent CNGs of *EGFR* in a TCGA glioblastoma multiforme cohort (~45%), and of *ERBB2* in nearly 14% of cases from a TCGA stomach cancer cohort. In summary, copy number changes of these apoptosis-related genes were highly frequent in certain cancer types, which may imply that apoptosis is critical in different cancer EMT processes.

### A connected biological map of EMT-implicated genes with concordance between CNG and increased gene expression

**Figure 4:**
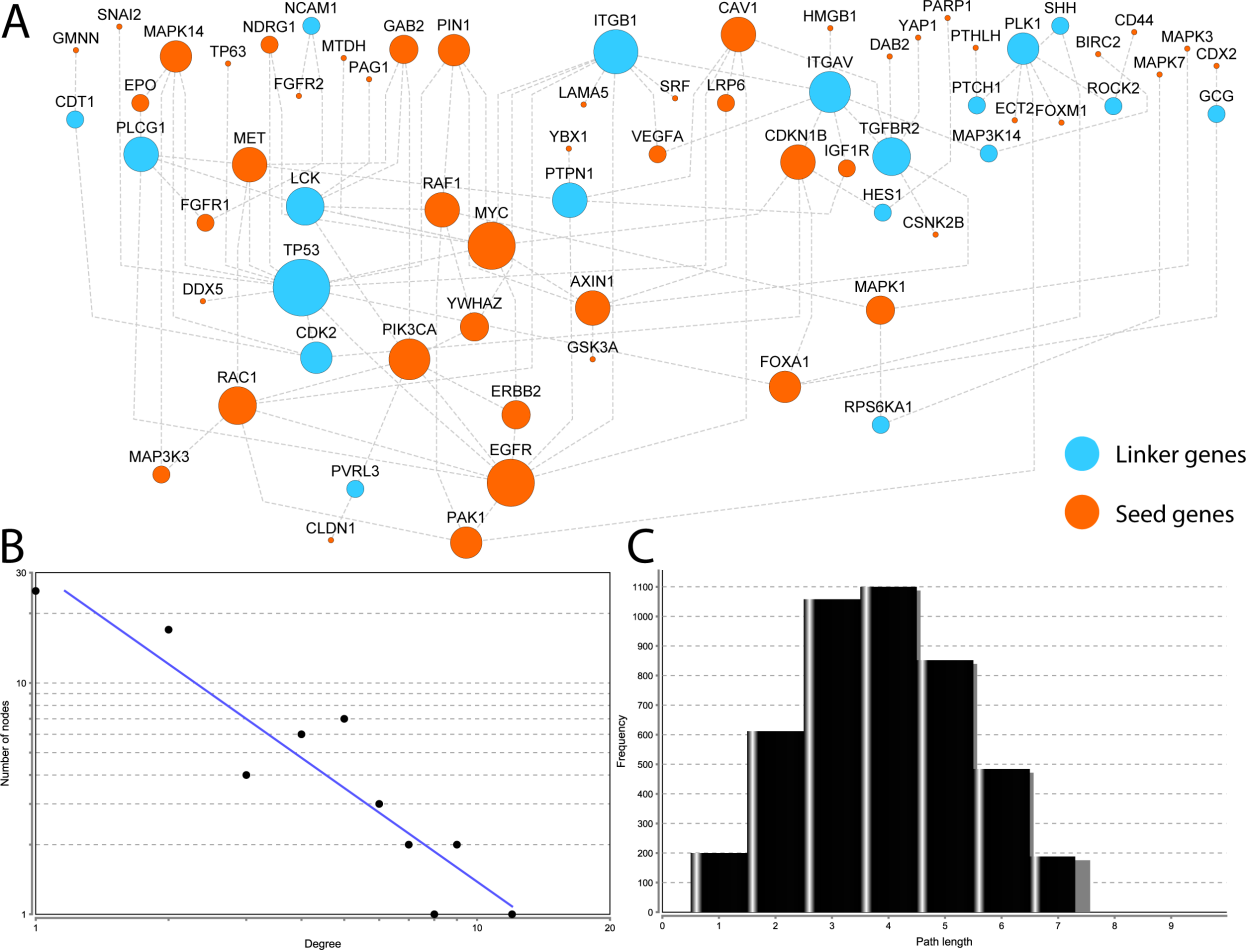
Reconstructed interaction map for EMT-implicated genes with CNGs and increased gene expression in matched tumor samples (**A**) The 49 genes in orange are those among the 71 EMT-implicated genes with increased expression induced by CNGs in 20 or more matched tumour samples. The other 19 genes in blue are linker genes that connect the 49 genes. The node size indicates the connection strength - the larger the node, the greater the degree of connectivity; The node size indicates the connection strength. The larger the node, the greater the degree of connectivity (**B**) the plot of degrees for all nodes in the network. The X axis represents the degrees of the nodes, and the Y axis represents the total number of nodes that correspond to the values on the X axis; The X axis represents the degree of the nodes, and the Y axis represents the total number of nodes that correspond to the values in the X axis (**C**) the plot of lengths for short paths in the network.

To demonstrate at a system level the shared cellular events related to the 71 EMT-implicated genes with increased expression caused by CNGs, we built a biological network using prepared pathway-based protein-protein interaction (PPI) data from the Pathway Commons database [17]. These reliable interactions are based on available evidences from known biological pathways, such as those recorded in the KEGG and Reactome pathway databases. Because these data avoid the high levels of noise, sparseness, and skewness that are often observed for physical interaction-based PPI networks. Using a module searching method described previously [18], we first mapped the 71 genes to the human pathway interactome. Then, we extracted a sub-network to connect as many of the input genes as possible. The final reconstructed network contains 68 genes with 100 links (Figure 4A). Of the 68 nodes, 49 are from our 71 input genes with concordant gene up-regulation and frequent CNGs. The remaining 19 nodes are linker genes that bridge the other 49 genes and form a fully connected cellular map. Notably, four of these linker genes are also related to EMT, namely *ITGB1*, *ROCK2*, *SHH*, and *TP53*. These four genes were not used as seeds because they did not exhibit concordant CNG and gene up-regulation in 20 or more matched tumor samples. Interestingly, *ITGB1* and *TP53* are connected with more than eight genes in the network, in which the the 8 highly-connected nodes can exchange information quickly.

The majority of genes in the reconstructed map are linked to each other in a highly modular structure according to their topological features. The degrees of all nodes in our reconstructed map follow a power law distribution *P(k)~k*^−b^, where *P(k)* is the probability that a gene has connections with *k* other genes and *b* is an exponent with an estimated value of 1.346 (Figure 4B). Thus the reconstructed map differs from the human interactome, in which the majority of genes are sparsely connected with a *b* exponent of 2.9 [19]. Moreover, the majority of the genes (~76.9%) could be reached within an average of three to five steps (Figure 4C). Both of these topological analyses indicate that the majority of genes in our map are connected with high modularity. Due to the tight connections, the highly-connected nodes in this network are critical for transducing biological information along the shortest paths. It is not surprising that TP53 is the most highly-connected node in the network (12 connections). *EGFR* and *MYC* follow *TP53*, with nine connections each. The other six highly-connected genes, having over five connections, are *ITGB1* (eight connections), *PIK3CA* (seven), *ITGAV* (seven), *TGFBR2* (six), *RAC1* (six), and *LCK* (six). In total, nine genes with six or more links, and all of them are associated with positive regulation of the phosphate metabolic process (GO:0045937, corrected *P*-value = 1.241E-9) and the regulation of phosphorylation (GO:0042325, corrected *P*-value = 8.013E-9). In addition, eight of the nine genes are related to a “pathway in cancer” (corrected *P*-value = 4.424E-9), wound healing (GO:0042060, corrected *P*-value = 8.013E-9), response to growth factors (GO:0070848, corrected *P*-value = 8.013E-9), abnormal extra-embryonic tissue morphology (corrected *P*-value = 1.587E-5), abnormal embryonic growth/weight/body size (corrected *P*-value = 4.224E-5), and embryonic lethality during organogenesis (corrected *P*-value = 4.580E-5). In summary, this reconstructed map for genes with potential CNG-driven gene up-regulation includesd multiple signaling pathways related to phosphorylation, which may be another molecular mechanism by which CNGs s up-regulate EMT-implicated genes.

### CNG-driven up-regulation in matched ovarian cancer samples

**Figure 5:**
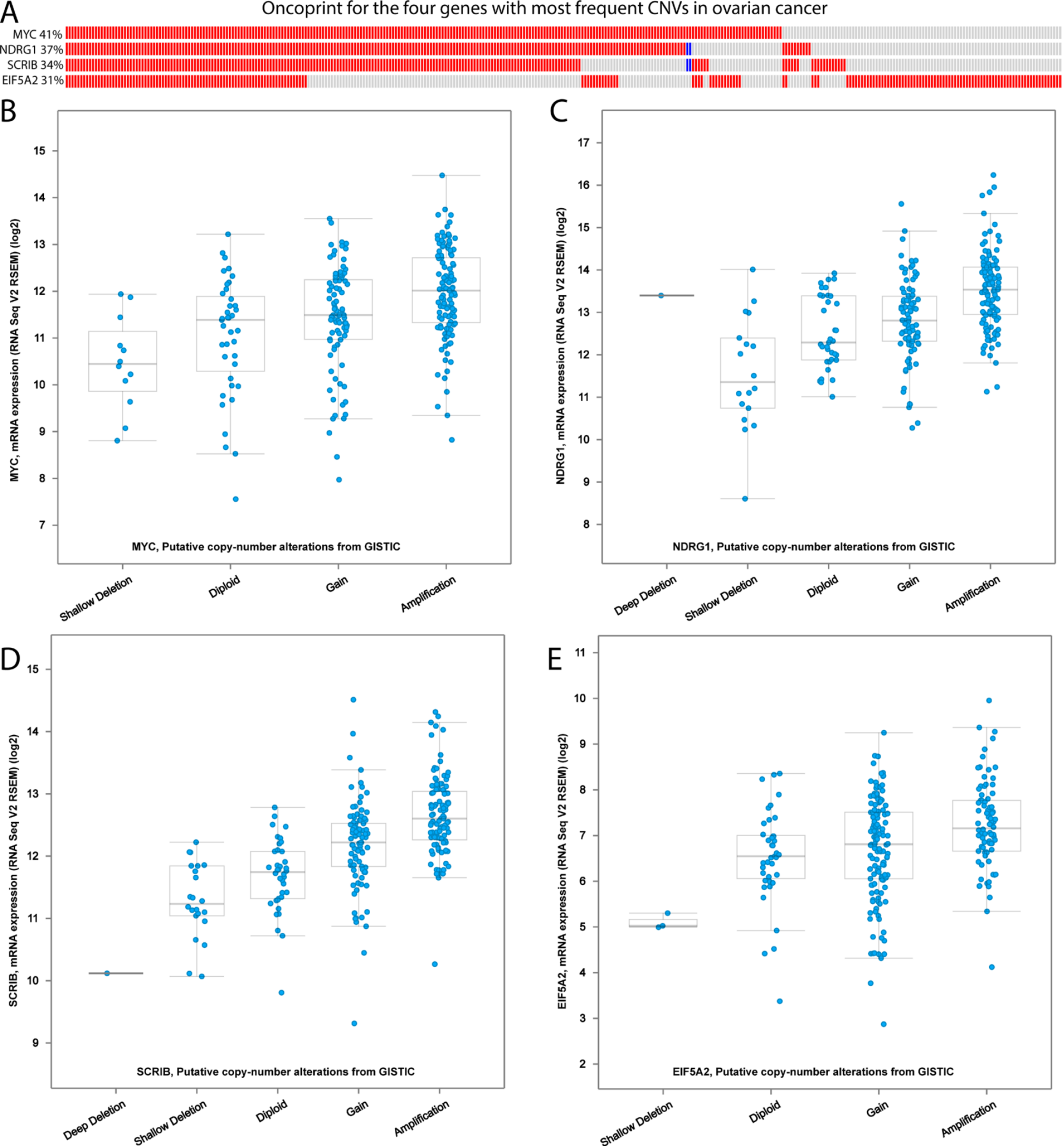
The correlation of CNVs and gene expression in the TCGA ovarian cancer cohort (**A**) The oncoprint for the four genes with the most frequent CNGs in the TCGA ovarian cancer cohort. The red cells represent a CNG in a tumour sample; the blue cells represent a CNL in the corresponding tumour sample. (**B–E**) The box plots for the four genes with the most frequent CNGs in the TCGA ovarian cancer cohort: (B) *MYC*, (C) *NDRG1*, (D) *SCRIB*, and (E) *EIF5A2*. These CNV levels are derived from the copy-number analysis algorithm GISTIC. For each gene, a deep loss is a copy-number level of “–2” with a possible ehomozygous deletion; a shallow loss is a copy-number level of “–1” with a possible heterozygous deletion; the normal gene copy number is noted as “diploid”; “gain” indicates a low level of CNG; and “amplification” indicates a high level of CNG.

To further explore the relationship between expression changes and CNGs, we focused on the TCGA ovarian cancer cohort. Among the 71 EMT-implicated genes with potential CNG-driven gene up-regulation, 70 genes exhibited at least one CNG in a patient. Next, we focused on the four genes with the most frequent CNGs in the ovarian cohort (CNGs detected in more than 30% of cases), namely *MYC*, *NDRG1*, *SCRIB*, and *EIF5A2*. The expression of these four genes was consistently high in those tumor samples with CNGs (Figure 5A–5E). When we compared the average gene expression between the CNG samples and the diploid samples, all of the results were significant (all four *P*-values < 0.05). We used *NDRG1,* a downstream tumor suppressor gene of the MYC signaling pathway in ovarian cancer [20–22], as an example. In our dbEMT, *NDRG1* was recorded as promoting the malignant progression of gastric cancer through EMT [23]. However, this gene is not reported to be involved in ovarian cancer, although it is associated with invasive potential in cervical and ovarian cancer cell lines [24]. Our results may imply that *NDRG1* promotes EMT in ovarian cancer, not just in cell lines. Overall, the systematic combination of gene expression and CNG data in ovarian cancer revealed that the gene dosage effects of CNGs in EMT-implicated genes may increase gene expression.

## DISCUSSION

This study revealed somatic CNV features of EMT-implicated genes in multiple cancer types, particularly with respect to the effects of CNGs on gene expression. EMT is a critical process for cancer metastasis, and large-scale gene copy gains in EMT-implicated genes may induce their up-regulation. Although previous studies have explored relationship between germline CNVs and gene expression [8], there have not been reports of direct links between somatic CNVs and gene expression dosage compensation. EMT can assist in the initiation of cancer metastasis [25], specifically, by disrupting the connections between cells in primary sites and thus enhancing their invasive properties. Therefore, it is not surprising that many of the 212 EMT-implicated genes with frequent CNGs encoded proteins located on the extracellular matrix (52 genes, GO:0005615, Corrected *P*-value = 1.36E-14) and cell surface (38 genes, GO:0009986, Corrected *P*-value = 2.41E-14).

It is worth noting that many of the 212 genes also functioned in “stem cell differentiation” (48 genes, GO:0048863, Corrected *P*-value = 1.01E-36). CNGs in these stem cell-related genes may invoke harmful stemness that promotes cancer cell proliferation [4]. Our results only provide the first insights into the correlation between EMT-implicated gene dosage and somatic CNVs. Additional systematic examinations of the expression of quantitative trait loci may provide more details concerning the relationship between CNVs and gene expression.

In this study, we used a large sample size across multiple cancer types to explore the patterns of CNV for EMT-implicated genes. The cohort size of each specific cancer type is still relatively small, on the level of hundreds of individuals. Thus, many low-frequency CNV events may not be detected for specific cancer types. A pan-cancer approach with a larger sample size may overcome this limitation and identify many novel genes that are mutated at high frequencies across cancer types. These highly recurrent CNVs may reveal overlooked driver genes for EMT.

One of the limitations of this study is the technology used for CNV detection in TCGA. TCGA primarily detects CNVs by using comparative genomic hybridization (CGH) arrays between matched tumor and normal samples, but this technique may miss signals outside pre-designed probes. Many of these undetected CNVs may also contribute to tumorigenesis. The other limitation of this study is that we only incorporated protein-coding gene expression and did not include non-coding gene expression. The further integration of large-scale CNV data and gene expression data for long non-coding RNAs may provide new insights into the roles of non-coding EMT-implicated genes [26].

According to the most recent CNV map of the human genome, an estimated 4.8–9.5% of the genome is subject to copy number change [27]. Despite the overwhelming occurrence of CNVs in the disease and health genome, it is still a challenge to estimate the extent to which CNVs contribute to disease-related phenotypic changes. Many genes may be completely deleted without any apparent phenotypic consequences [27]. The term “gene dosage” refers to the exact number of copies of a particular gene in a genome [8]. However, it is widely acknowledged that the amount of gene product produced in a cell depends more on transcriptional regulation than on copy number. Our study on ovarian cancer may imply that the gene dosage is related to the amount of gene product that the cell is able to express. Such changes in gene dosage may have significant phenotypic consequences during EMT.

Although the majority of CNVs may not be directly linked to tumorigenesis, they may activate gene expression, according to our results. These gene expression changes may promote cancer progression through broader gene-gene interactions. For example, CNVs in genes encoding tumor suppressors and oncogenic transcription factors/microRNAs may have more profound effects on their target genes than on these genes themselves.

In conclusion, our systematic survey of the relationship between CNVs and gene expression in EMT further supports the evidence that gene dosage correlates with gene expression. We found concordance between CNG and gene up-regulation for numerous EMT-related genes in hundreds of tumor samples from different cancer types.

## MATERIALS AND METHODS

### The curated EMT-implicated genes from the dbEMT

To systematically study EMT-implicated genes, we performed an extensive literature search followed and then manually assembled the data. We focused our literature search on CNV studies, gene expression-based functional studies, genome-wide association studies and relevant non-coding RNA analyses using the following expression against the PubMed database: (“Epithelial-mesenchymal transition” [Title/Abstract] OR “Epithelial mesenchymal transition” [Title/Abstract] OR “EMT” [Title/Abstract]) AND ((“genome-wide association study” [Title/Abstract] OR “genome wide association study” [Title/Abstract]) OR (“gene” [Title/Abstract] AND (“association” [Title/Abstract] OR “microarray” [Title/Abstract] OR “expression” [Title/Abstract] OR “linkage” [Title/Abstract] OR “proteomics” [Title/Abstract] OR “genetic” [Title/Abstract] OR “metabolomics” [Title/Abstract] OR “copy number variation” [Title/Abstract] OR “idiopathic” [Title/Abstract] OR “hereditable” [Title/Abstract] OR “family” [Title/Abstract] OR “mouse model” [Title/Abstract] OR “animal model” [Title/Abstract] OR “microRNA” [Title/Abstract] OR “mutation” [Title/Abstract] OR “SNP” [Title/Abstract] OR “drug” [Title/Abstract] OR “transporter” [Title/Abstract]))). This search returned 1507 abstracts on 19 December 2013. We manually curated the experimentally verified candidate genes. This information and the related annotations were stored in the dbEMT database and published for public use. In this study, we downloaded all 377 curated human EMT-implicated genes from the dbEMT database in plain text with all the official symbols (http://dbEMT.bioinfo-minzhao.org/download.cgi) [10].

To align the EMT-implicated genes with reported CNVs, we first annotated all the genes with precise genomic locations. To this end, we downloaded the RefSeq database from NCBI (ftp://ftp.ncbi.nlm.nih.gov/gene/DATA/gene2refseq.gz). Then, we implemented a Perl script to extract all the genomic coordinates of the 377 EMT-implicated genes from the completed genomic sequences. To provide the most up-to-date information, we used genome version GRCH 38 for accurate genomic locations.

### Classification of copy number gain and loss in TCGA pan-cancer CNV data

TCGA CNV data were downloaded from COSMIC [28] (V73, https://cancer.sanger.ac.uk/cosmic/files?data=/files/grch38/cosmic/v73/CosmicCompleteCNA.tsv.gz) so that the CNVs with precise gain and loss information could be collected. A number of criteria were used by COSMIC to define copy number loss and gain. For CNGs in the TCGA pan-cancer study, the following parameters were required: (average genome ploidy < = 2.7 AND total DNA segment copy number > = 5) OR (average genome ploidy > 2.7 AND total DNA segment copy number > = 9). Similarly, the criteria for CNL were: (average genome ploidy < =2.7 AND total DNA segment copy number = 0) OR (average genome ploidy > 2.7 AND total DNA segment copy number > = (average genome ploidy – 2.7)). In this study, we adopted the same COSMIC criteria and aligned all of the CNV regions with EMT-implicated genes using the GRCH 38 coordinates. We annotated a total of 365 EMT-implicated genes with precise overlapping CNG and CNL information. To cross-validate the data among different cancers, we counted the number of CNG and CNL samples regardless of cancer type. To explore the oncogenic role of EMT-implicated genes, we collected those EMT-implicated genes with more CNGs than CNLs. A threshold was set to collect those genes for which the number of tumor samples with CNGs was at least twice the number of samples with CNLs. Ultimately, 212 EMT-implicated genes with more CNGs than CNLs were collected for the subsequent integrative gene expression analysis.

### Matching of gene expression changes and CNVs for tumor samples

To investigate CNV-driven gene expression changes for EMT-implicated genes, we downloaded TCGA pan-cancer gene expression data from the COSMIC database (Version 73). Here, we focused only on those gene expression changes in TCGA samples with matched EMT-related CNGs. For gene expression quantification, the RSEM quantification results from the RNAseq V2 platform in COSMIC were used. The averages and standard deviations of the expression values were calculated based on tumor samples that were diploid for each corresponding gene.

The standard Z-score was used to characterize whether an EMT-implicated gene was over- or under-expressed, similar to its implementation in the COSMIC database [28]. Here, Z-score is a standardized variables based on a transformation of the *P*-value calculated using the formula as below:

$$Z=x-\frac{\mu }{\sigma }$$

where *x* is the gene expression of a gene in the individual sample; μ is the averaged expression score of a gene across multiple TCGA samples; and σ is the standard deviation of the expression scores of the gene in different individual samples. The threshold of a Z-score ≥ 2 was used to identify EMT-implicated genes of interest among the samples. A Z-score greater than 2 was defined as over-expression, which corresponds to a *P*-value less than 0.01.

For the 71 EMT-implicated genes with concordance between CNG and up-regulation, we further systematically examined the pan-cancer somatic CNV patterns in the TCGA samples using the cBio portal [29]. In addition, we focused on samples with both expression changes and CNVs in the ovarian cancer cohort from TCGA to verify whether the CNGs in EMT-implicated genes correlated with increased gene expression.

### Sub-network extraction for EMT-implicated genes with concordance between CNGs and up-regulation

To reveal the global gene-gene interaction among genes with frequent CNGs and consistent gene up-regulation, we extracted a sub-network from the human interactome. First, we collected a non-redundant human interactome from the PathwayCommons database [17, 30] with 3, 629 proteins and 36, 034 protein-protein interactions. We only used interactions from well-curated pathway databases (HumanCyc, Reactome, and the KEGG pathway [31]). Thus, the interactome consisted of links with biological meaning rather than physical interactions. From these pathway-based interactions, we extracted a sub-network using an approach similar to the one we implemented in previous studies [18, 30]. In this sub-network extraction strategy, all 71 of the EMT-implicated genes were mapped onto the human pathway-based interactome as seeds. A greedy module search was used to identify the sub-network with as many seed genes as possible. Finally, the sub-network with the most EMT-implicated genes and the shortest connecting paths was formed. We further characterized the basic topological properties and overall function of the sub-network using the NetworkAnalyzer plug-in in Cytoscape 2.8 (Figure 4B–4C) [32]. The degree is defined as the total number of links for each node in the network [33]. The network layout was based on Cytoscape 2.8 [32] (Figure 4A).

## SUPPLEMENTARY MATERIALS TABLES





## Abbreviations

TCGA: The Cancer Genome Atlas
EMT: Epithelial-Mesenchymal Transition
CNV: Copy Number Variation
CNG: Copy Number Gain
CNL: Copy Number Loss.
