# Supplementary material for: Expression of epithelial-mesenchymal transition-related genes increases with copy number in multiple cancer types
Source: Oncotarget. 2016 Mar 25;7(17):24688–99. doi: 10.18632/oncotarget.8371 (PMC5029734; doi:10.18632/oncotarget.8371)
Supplement: Supplementary file 1 [file oncotarget-07-24688-s001.pdf]

Expression of epithelial-mesenchymal transition-related genes increases with copy number in multiple cancer types

Supplementary Materials

Supplementary Table S1: The 212 EMT-implicated genes with frequent CNGs

GeneID	Symbol	# of samples with copy number loss	# of samples with copy number gain	Ratio for the samples with CNL and CNG
100133941	CD24	24	84	3.5
10049	DNAJB6	7	76	10.857
1027	CDKN1B	22	92	4.182
10397	NDRG1	1	542	542
10413	YAP1	19	122	6.421
1045	CDX2	22	101	4.591
10631	POSTN	27	80	2.963
10637	LEFTY1	1	165	165
10657	KHDRBS1	4	18	4.5
1295	COL8A1	\	68	11.333
1296	COL8A2	9	28	3.111
1316	KLF6	17	55	3.235
1364	CLDN4	2	52	26
142	PARP1	1	168	168
1432	MAPK14	2	39	19.5
1460	CSNK2B	1	40	40
1500	CTNND1	2	19	9.5
152007	GLIPR2	6	55	9.167
1522	CTSZ	3	249	83
1523	CUX1	8	109	13.625
1601	DAB2	3	244	81.333
1655	DDX5	6	90	15
1748	DLX4	4	79	19.75
177	AGER	2	33	16.5
182	JAG1	14	96	6.857
1839	HBEGF	8	20	2.5
1894	ECT2	2	437	218.5
1906	EDN1	5	75	15
1909	EDNRA	12	24	2
1956	EGFR	5	646	129.2
1958	EGR1	7	16	2.286
2001	ELF5	5	53	10.6
2022	ENG	5	17	3.4

2034	EPAS1	3	34	11.333
2056	EPO	5	110	22
2064	ERBB2	3	275	91.667
2077	ERF	7	35	5
2118	ETV4	6	16	2.667
2146	EZH2	10	83	8.3
220001	VWCE	3	17	5.667
2260	FGFR1	43	327	7.605
2263	FGFR2	15	42	2.8
2274	FHL2	2	17	8.5
22822	PHLDA1	8	37	4.625
2296	FOXC1	18	50	2.778
2305	FOXM1	10	129	12.9
23129	PLXND1	7	59	8.429
2321	FLT1	24	98	4.083
23513	SCRIB	1	279	279
25794	FSCN2	5	44	8.8
25875	LETMD1	2	22	11
25937	WWTR1	5	236	47.2
2625	GATA3	16	63	3.938
269	AMHR2	3	14	4.667
27076	LYPD3	5	46	9.2
28984	C13orf15	37	83	2.243
290	ANPEP	15	49	3.267
29126	CD274	48	104	2.167
2931	GSK3A	7	34	4.857
2932	GSK3B	9	71	7.889
2934	GSN	6	12	2
301	ANXA1	3	19	6.333
3037	HAS2	3	588	196
3082	HGF	4	111	27.75
3146	HMGB1	25	94	3.76
3169	FOXA1	11	102	9.273
3206	HOXA10	2	123	61.5
3217	HOXB7	4	66	16.5
3219	HOXB9	4	69	17.25
329	BIRC2	14	114	8.143
3315	HSPB1	3	49	16.333
3398	ID2	3	26	8.667
3418	IDH2	14	46	3.286
3480	IGF1R	23	72	3.130
3486	IGFBP3	1	85	85
3491	CYR61	9	18	2
3553	IL1B	3	19	6.333
3655	ITGA6	2	33	16.5

3678	ITGA5	2	26	13
3688	ITGB1	7	37	5.286
3690	ITGB3	9	25	2.778
3691	ITGB4	5	73	14.6
3756	KCNH1	3	171	57
3815	KIT	6	104	17.333
3845	KRAS	6	237	39.5
3880	KRT19	6	42	7
3911	LAMA5	3	202	67.333
3952	LEP	6	86	14.333
3987	LIMS1	2	15	7.5
4040	LRP6	17	111	6.529
406928	MIR137	9	23	2.556
406949	MIR15B	2	245	122.5
406969	MIR194-1	2	173	86.5
406985	MIR200C	8	104	13
406991	MIR21	5	142	28.4
407010	MIR23A	6	80	13.333
4072	EPCAM	4	32	8
4093	SMAD9	32	83	2.594
4192	MDK	5	23	4.6
4215	MAP3K3	6	109	18.167
4233	MET	8	145	18.125
4314	MMP3	15	88	5.867
4316	MMP7	15	114	7.6
4322	MMP13	15	85	5.667
4323	MMP14	4	48	12
4585	MUC4	5	285	57
4609	MYC	1	784	784
4613	MYCN	3	49	16.333
4853	NOTCH2	4	22	5.5
4904	YBX1	5	45	9
4920	ROR2	6	13	2.167
4921	DDR2	1	192	192
5052	PRDX1	5	20	4
5058	PAK1	9	184	20.444
51053	GMNN	5	64	12.8
5141	PDE4A	7	63	9
51474	LIMA1	3	20	6.667
5290	PIK3CA	12	438	36.5
5300	PIN1	7	56	8
5329	PLAUR	5	48	9.6
5460	POU5F1	1	40	40
5468	PPARG	11	43	3.909
54810	GIPC2	10	26	2.6

54845	ESRP1	3	428	142.667
54910	SEMA4C	3	24	8
5578	PRKCA	12	143	11.917
55818	KDM3A	3	14	4.667
5581	PRKCE	14	48	3.429
55824	PAG1	1	418	418
558	AXL	5	39	7.8
5594	MAPK1	15	32	2.133
5595	MAPK3	2	45	22.5
5598	MAPK7	23	48	2.087
5652	PRSS8	2	47	23.5
5653	KLK6	7	28	4
56648	EIF5A2	2	425	212.5
57057	TBX20	5	103	20.6
57118	CAMK1D	22	82	3.727
5743	PTGS2	3	216	72
5744	PTHLH	8	155	19.375
5747	PTK2	5	500	100
57496	MKL2	3	52	17.333
5764	PTN	9	99	11
57669	EPB41L5	5	28	5.6
5784	PTPN14	10	170	17
5803	PTPRZ1	6	99	16.5
5879	RAC1	10	103	10.3
5894	RAF1	12	33	2.75
6093	ROCK1	10	24	2.4
6374	CXCL5	15	54	3.6
6382	SDC1	2	27	13.5
6469	SHH	18	138	7.667
648	BMI1	20	41	2.05
6493	SIM2	7	27	3.857
650	BMP2	12	74	6.167
652	BMP4	6	31	5.167
6591	SNAI2	4	205	51.25
6624	FSCN1	8	97	12.125
6651	SON	3	12	4
6662	SOX9	7	62	8.857
6667	SP1	3	14	4.667
6722	SRF	3	91	30.333
673	BRAF	8	104	13
6774	STAT3	5	19	3.8
6776	STAT5A	5	19	3.8
6777	STAT5B	5	19	3.8
6868	ADAM17	3	29	9.667
6909	TBX2	10	132	13.2

6926	TBX3	5	16	3.2
6935	ZEB1	9	33	3.667
7003	TEAD1	12	26	2.167
7024	TFCP2	3	24	8
7039	TGFA	2	21	10.5
7040	TGFB1	4	35	8.75
7041	TGFB1I1	3	47	15.667
7042	TGFB2	2	173	86.5
7049	TGFBR3	12	30	2.5
7056	THBD	9	51	5.667
7124	TNF	2	38	19
7227	TRPS1	4	594	148.5
7291	TWIST1	12	148	12.333
7421	VDR	5	46	9.2
7422	VEGFA	3	105	35
7431	VIM	9	38	4.222
7447	VSNL1	3	32	10.667
7448	VTN	1	64	64
7471	WNT1	4	22	5.5
7490	WT1	10	50	5
7534	YWHAZ	1	495	495
7764	ZNF217	1	335	335
7791	ZYX	7	73	10.429
7852	CXCR4	5	10	2
8061	FOSL1	7	34	4.857
8312	AXIN1	20	51	2.55
8313	AXIN2	9	94	10.444
84695	LOXL3	2	17	8.5
8487	GEMIN2	6	65	10.833
857	CAV1	8	123	15.375
8626	TP63	30	394	13.133
8668	EIF3I	5	17	3.4
873	CBR1	9	18	2
89780	WNT3A	7	162	23.143
9076	CLDN1	8	336	42
90	ACVR1	3	20	6.667
92140	MTDH	1	423	423
9241	NOG	5	82	16.4
9372	ZFYVE9	7	17	2.429
94025	MUC16	30	61	2.033
9475	ROCK2	2	32	16
960	CD44	6	76	12.667
9723	SEMA3E	7	126	18
9846	GAB2	6	185	30.833

Supplementary Table S2: The biological pathways enriched for the 212 EMT-implicated genes with frequent CNGs

Name	*p*-value	*q*-value FDR B & Y	Hit Count in Query List	Hit in Query List
Proteoglycans in cancer	4.24E-28	5.78E-25	39	MYC,HGF,ERBB2,ITGA5,ITGB1,ITGB3,PTK2,STAT3,DDX5,TWIST1,MET,BRAF,PIK3CA,WNT3A,PLAUR,FGFR1,SDC1,RAC1,VEGFA,ROCK2,KRAS,RAF1,VTN,HBEGF,WNT1,CAV1,TGFB1,TGFB2,MAPK14,IGF1R,EGFR,CD44,PAK1,MIR21,PRKCA,ROCK1,TNF,MAPK1,MAPK3
Pathways in cancer	7.23E-24	4.93E-21	41	MYC,CDKN1B,HGF,ERBB2,ITGA6,ITGB1,PTGS2,PTK2,STAT3,STAT5A,AXIN1,STAT5B,AXIN2,MET,BMP2,BMP4,BRAF,PIK3CA,WNT3A,FGFR1,FGFR2,KIT,RAC1,VEGFA,KRAS,RAF1,WNT1,SHH,LAMA5,BIRC2,PPARG,GSK3B,TGFA,TGFB1,TGFB2,IGF1R,EGFR,PRKCA,MAPK1,MAPK3,EPAS1
Integrated Pancreatic Cancer Pathway	3.99E-19	4.93E-21	29	MYC,CDKN1B,SP1,ERBB2,EZH2,PTGS2,STAT5A,PIK3CA,FGFR1,RAC1,VEGFA,KRAS,RAF1,ANXA1,WT1,SHH,GSK3A,TGFB1,LEFTY1,MAPK14,IGFBP3,EGFR,EGR1,PAK1,PRKCA,TNF,MAPK1,MAPK3,MAPK7
MicroRNAs in cancer	1.34E-18	4.56E-16	34	MYC,CDKN1B,ERBB2,ITGA5,EZH2,ITGB3,PTGS2,STAT3,BMI1,MET,PIK3CA,WNT3A,NOTCH2,VEGFA,KRAS,RAF1,VIM,ZEB1,TGFB2,MIR137,MIR15B,EGFR,TP63,MIR194-1,CD44,MIR21,PRKCA,PRKCE,ROCK1,MIR200C,MAPK1,MAPK7,FSCN1,MIR23A
Focal adhesion	2.18E-18	5.94E-16	29	HGF,ERBB2,ITGA6,ITGA5,ITGB1,ITGB3,ITGB4,ZYX,PTK2,MET,BRAF,PIK3CA,RAC1,VEGFA,ROCK2,RAF1,FLT1,VTN,LAMA5,BIRC2,CAV1,GSK3B,IGF1R,EGFR,PAK1,PRKCA,ROCK1,MAPK1,MAPK3
MicroRNAs in cardiomyocyte hypertrophy	2.04E-16	3.98E-14	21	STAT3,PIK3CA,WNT3A,FGFR2,RAC1,ROCK2,RAF1,EDN1,GSK3B,TGFB1,MAPK14,IGF1R,MIR15B,LRP6,MIR21,ROCK1,TNF,MAPK1,MAPK3,MAPK7,MIR23A
Prolactin Signaling Pathway	9.55E-15	1.58E-12	17	MYC,ERBB2,ITGB1,PTK2,STAT3,GAB2,STAT5A,STAT5B,PIK3CA,RAC1,RAF1,YWHAZ,GSK3B,MAPK14,PAK1,MAPK1,MAPK3
ErbB signaling pathway	1.05E-14	1.58E-12	18	MYC,CDKN1B,ERBB2,PTK2,STAT5A,STAT5B,BRAF,PIK3CA,KRAS,RAF1,HBEGF,GSK3B,TGFA,EGFR,PAK1,PRKCA,MAPK1,MAPK3
IL-3 Signaling Pathway	1.66E-14	2.26E-12	19	PTK2,STAT3,GAB2,STAT5A,STAT5B,PIK3CA,HSPB1,RAC1,KRAS,RAF1,YWHAZ,GSK3A,GSK3B,MAPK14,PAK1,PRKCA,MAPK1,MAPK3,MAPK7
AGE/RAGE pathway	1.92E-14	2.38E-12	16	SP1,STAT3,STAT5A,STAT5B,AGER,MMP7,MMP13,MMP14,RAC1,RAF1,MAPK14,EGFR,PRKCA,ROCK1,MAPK1,MAPK3
TGF-beta signaling pathway	3.02E-14	3.43E-12	17	MYC,SP1,NOG,ACVR1,BMP2,BMP4,ZFYVE9,AMHR2,ID2,TGFB1,TGFB2,LEFTY1,ROCK1,TNF,MAPK1,MAPK3,SMAD9
EGF receptor signaling pathway	5.78E-14	6.06E-12	19	ERBB2,STAT3,GAB2,MAP3K3,STAT5A,STAT5B,BRAF,PIK3CA,RAC1,RAF1,HBEGF,YWHAZ,TGFA,MAPK14,EGFR,PRKCA,PRKCE,MAPK1,MAPK3

Signaling by FGFR in disease	3.11E-13	2.94E-11	22	CDKN1B,ERBB2,STAT3,GAB2,STAT5A,STAT5B,BRAF,PIK3CA,FGFR1,FGFR2,KIT,KRAS,RAF1,HBEGF,GSK3A,GSK3B,EGFR,PRKCA,PRKCE,MAPK1,MAPK3,CUX1
Signaling by SCF-KIT	3.24E-13	2.94E-11	20	CDKN1B,ERBB2,STAT3,GAB2,STAT5A,STAT5B,PIK3CA,FGFR1,FGFR2,KIT,RAC1,KRAS,RAF1,HBEGF,GSK3A,GSK3B,EGFR,PRKCA,MAPK1,MAPK3
Renal cell carcinoma	3.63E-13	3.09E-11	15	HGF,MET,BRAF,PIK3CA,RAC1,VEGFA,KRAS,RAF1,TGFA,TGFB1,TGFB2,PAK1,MAPK1,MAPK3,EPAS1
Angiogenesis	1.61E-12	1.29E-10	20	PTK2,STAT3,AXIN1,AXIN2,BRAF,PIK3CA,JAG1,FGFR1,NOTCH2,VEGFA,RAF1,WNT1,GSK3B,TGFB1I1,MAPK14,PAK1,PRKCA,PRKCE,MAPK1,MAPK3
PI3K-Akt signaling pathway	2.19E-12	1.65E-10	29	MYC,CDKN1B,EPO,HGF,ITGA6,ITGA5,ITGB1,ITGB3,ITGB4,PTK2,MET,PIK3CA,FGFR1,FGFR2,KIT,RAC1,VEGFA,KRAS,RAF1,FLT1,VTN,LAMA5,YWHAZ,GSK3B,IGF1R,EGFR,PRKCA,MAPK1,MAPK3
Oncostatin M Signaling Pathway	3.18E-12	2.28E-10	14	CDKN1B,STAT3,STAT5B,MMP3,MMP13,KRAS,RAF1,MAPK14,CYR61,EGR1,PRKCA,PRKCE,MAPK1,MAPK3
Spinal Cord Injury	9.79E-12	6.35E-10	17	MYC,CDKN1B,SOX9,PTPRZ1,RAC1,ROCK2,VIM,ANXA1,LEP,TGFB1,EGFR,EGR1,PRKCA,TNF,MAPK1,MAPK3,IL1B
Alpha6-Beta4 Integrin Signaling Pathway	1.47E-11	8.65E-10	14	ERBB2,ITGA6,ITGB4,PTK2,MET,PIK3CA,MMP7,RAC1,VIM,LAMA5,YWHAZ,EGFR,PAK1,PRKCA
Wnt Signaling Pathway NetPath	1.52E-11	8.65E-10	17	SOX9,DAB2,AXIN1,AXIN2,PIN1,WNT3A,FHL2,RAC1,RAF1,WNT1,ROR2,GSK3B,CSNK2B,LRP6,PRKCA,MAPK1,MAPK3
Integrin-mediated cell adhesion	1.52E-11	8.65E-10	16	ITGA6,ITGA5,ITGB1,ITGB3,ITGB4,ZYX,PTK2,BRAF,RAC1,ROCK2,RAF1,CAV1,PAK1,ROCK1,MAPK1,MAPK7
Downstream signal transduction	3.05E-11	1.66E-9	19	CDKN1B,ERBB2,STAT3,STAT5A,STAT5B,PIK3CA,FGFR1,FGFR2,KIT,KRAS,RAF1,HBEGF,GSK3A,GSK3B,EGFR,PRKCA,PRKCE,MAPK1,MAPK3
EGFR1 Signaling Pathway	3.60E-11	1.89E-9	20	MYC,SP1,STAT3,GAB2,MAP3K3,STAT5A,STAT5B,PIK3CA,RAC1,KRAS,RAF1,CAV1,MAPK14,EGFR,PAK1,PRKCA,MAPK1,MAPK3,CTNND1,MAPK7
Colorectal cancer	4.28E-11	2.16E-9	13	MYC,AXIN1,AXIN2,BRAF,PIK3CA,RAC1,KRAS,RAF1,GSK3B,TGFB1,TGFB2,MAPK1,MAPK3
Senescence and Autophagy	5.17E-11	2.50E-9	16	CDKN1B,BMI1,BMP2,BRAF,MMP14,RAF1,VTN,GSK3B,GSN,TGFB1,MAPK14,IGF1R,IGFBP3,CD44,MAPK1,IL1B
Signaling events mediated by VEGFR1 and VEGFR2	5.31E-11	2.50E-9	13	ITGB3,PIK3CA,HSPB1,RAC1,VEGFA,FLT1,CAV1,PAK1,PRKCA,ROCK1,MAPK1,MAPK3,EPAS1
Endometrial cancer	7.37E-11	3.35E-9	12	MYC,ERBB2,AXIN1,AXIN2,BRAF,PIK3CA,KRAS,RAF1,GSK3B,EGFR,MAPK1,MAPK3
PDGF signaling pathway	7.71E-11	3.39E-9	17	MYC,ERF,SRF,STAT3,GAB2,STAT5A,STAT5B,BRAF,PIK3CA,RAF1,GSK3A,GSK3B,PRKCA,ELF5,MAPK1,MAPK3,MAPK7
Cardiac Progenitor Differentiation	9.39E-11	4.00E-9	12	NOG,BMP4,CXCR4,WNT3A,TBX20,KIT,ANPEP,ROR2,POU5F1,GSK3B,TGFB1,MAPK14

Signaling Pathways in Glioblastoma	9.86E-11	4.07E-9	14	CDKN1B,ERBB2,MET,BRAF,PIK3CA,FGFR1,FGFR2,KRAS,RAF1,IGF1R,EGFR,PRKCA,MAPK1,MAPK3
Signaling by ERBB4	1.19E-10	4.77E-9	18	CDKN1B,ERBB2,STAT5A,STAT5B,PIK3CA,YAP1,FGFR1,ADAM17,FGFR2,KIT,KRAS,RAF1,HBEGF,GSK3A,GSK3B,EGFR,MAPK1,MAPK3
Ras Pathway	1.47E-10	5.47E-9	13	SRF,STAT3,BRAF,PIK3CA,RAC1,KRAS,RAF1,GSK3A,GSK3B,MAPK14,PAK1,MAPK1,MAPK3
ErbB signaling pathway	1.50E-10	5.47E-9	12	MYC,CDKN1B,ERBB2,PTK2,STAT5A,KRAS,HBEGF,GSK3B,TGFA,EGFR,PRKCA,MAPK1
TGF Beta Signaling Pathway	1.50E-10	5.47E-9	12	NOG,STAT3,BMP4,ZFYVE9,WNT1,TGFB1,TGFBR3,LEFTY1,TNF,MAPK3,ENG,SMAD9
Rap1 signaling pathway	1.53E-10	5.47E-9	21	HGF,ITGB1,ITGB3,MET,BRAF,PIK3CA,FGFR1,FGFR2,KIT,RAC1,VEGFA,KRAS,RAF1,FLT1,MAPK14,IGF1R,EGFR,PRKCA,MAPK1,MAPK3,CTNND1
Syndecan-1-mediated signaling events	1.76E-10	6.09E-9	8	HGF,MET,MMP7,SDC1,LAMA5,TGFB1,MAPK1,MAPK3
IL-5 Signaling Pathway	1.79E-10	6.09E-9	13	CDKN1B,STAT3,STAT5A,STAT5B,RAC1,KRAS,RAF1,YWHAZ,GSK3A,GSK3B,MAPK14,MAPK1,MAPK3
Hippo signaling pathway	2.07E-10	6.71E-9	18	MYC,AXIN1,AXIN2,BMP2,BMP4,YAP1,WNT3A,WNT1,ID2,BIRC2,WWTR1,TEAD1,YWHAZ,GSK3B,TGFB1,TGFB2,SNAI2,SCRIB
TGF-beta Receptor Signaling Pathway	2.07E-10	6.71E-9	18	MYC,SP1,DAB2,AXIN1,AXIN2,ZFYVE9,YAP1,VDR,ZEB1,CAV1,TGFB1,TGFB2,TGFBR3,MAPK14,CD44,ROCK1,EIF3I,ENG
Extracellular matrix organization	2.35E-10	7.45E-9	23	ITGA6,ITGA5,ITGB1,ITGB3,ITGB4,BMP2,BMP4,ADAM17,LOXL3,MMP3,MMP7,MMP13,MMP14,SDC1,COL8A1,COL8A2,VTN,DDR2,LAMA5,TGFB1,TGFB2,CD44,PRKCA
Signal transduction by L1	2.96E-10	9.18E-9	10	ITGA5,ITGB1,ITGB3,FGFR1,RAC1,EGFR,CSNK2B,PAK1,MAPK1,MAPK3
Integrins in angiogenesis	3.09E-10	9.36E-9	11	CDKN1B,ITGB3,PIK3CA,SDC1,RAC1,VEGFA,VTN,IGF1R,ROCK1,MAPK1,MAPK3
Signaling by PDGF	3.40E-10	1.01E-8	19	CDKN1B,ERBB2,STAT3,STAT5A,STAT5B,PIK3CA,FGFR1,FGFR2,KIT,KRAS,RAF1,HBEGF,GSK3A,GSK3B,EGFR,PRKCA,PRKCE,MAPK1,MAPK3
NGF signalling via TRKA from the plasma membrane	3.64E-10	1.06E-8	20	CDKN1B,ERBB2,STAT3,BRAF,PIK3CA,FGFR1,FGFR2,KIT,KRAS,RAF1,HBEGF,GSK3A,GSK3B,MAPK14,EGFR,PRKCA,PRKCE,MAPK1,MAPK3,MAPK7
Chronic myeloid leukemia	3.75E-10	1.07E-8	13	MYC,CDKN1B,GAB2,STAT5A,STAT5B,BRAF,PIK3CA,KRAS,RAF1,TGFB1,TGFB2,MAPK1,MAPK3
Prostate cancer	4.34E-10	1.21E-8	14	CDKN1B,ERBB2,BRAF,PIK3CA,FGFR1,FGFR2,KRAS,RAF1,GSK3B,TGFA,IGF1R,EGFR,MAPK1,MAPK3
Angiopoietin receptor Tie2-mediated signaling	5.08E-10	1.38E-8	11	ITGA5,ITGB1,STAT5A,STAT5B,PIK3CA,RAC1,MAPK14,PAK1,TNF,MAPK1,MAPK3
VEGF signaling pathway	5.42E-10	1.40E-8	12	PTGS2,PTK2,PIK3CA,HSPB1,RAC1,VEGFA,KRAS,RAF1,MAPK14,PRKCA,MAPK1,MAPK3
ALK in cardiac myocytes	5.45E-10	1.40E-8	10	NOG,ACVR1,AXIN1,BMP2,BMP4,WNT1,GSK3B,TGFB1,TGFB2,TGFBR3
Signaling of Hepatocyte Growth Factor Receptor	5.45E-10	1.40E-8	10	HGF,ITGB1,PTK2,STAT3,MET,PIK3CA,RAF1,PAK1,MAPK1,MAPK3

Syndecan interactions	8.70E-10	2.20E-8	8	ITGA6,ITGB1,ITGB3,ITGB4,SDC1,VTN,TGFB1,PRKCA
Signalling by NGF	9.99E-10	2.48E-8	23	CDKN1B,ERBB2,STAT3,BRAF,PIK3CA,FGFR1,ADAM17,FGFR2,KIT,RAC1,KRAS,RAF1,HBEGF,ECT2,GSK3A,GSK3B,MAPK14,EGFR,PRKCA,PRKCE,MAPK1,MAPK3,MAPK7
Regulation of actin cytoskeleton	1.21E-9	2.94E-8	20	ITGA6,ITGA5,ITGB1,ITGB3,ITGB4,PTK2,BRAF,PIK3CA,FGFR1,FGFR2,RAC1,ROCK2,KRAS,RAF1,GSN,EGFR,PAK1,ROCK1,MAPK1,MAPK3
IL2-mediated signaling events	1.28E-9	3.05E-8	11	MYC,STAT3,GAB2,STAT5A,STAT5B,PIK3CA,RAF1,MAPK14,PRKCE,MAPK1,MAPK3
Adipogenesis	1.43E-9	3.36E-8	16	SP1,GATA3,STAT3,STAT5A,STAT5B,TWIST1,BMP2,BMP4,KLF6,WNT1,WWTR1,PPARG,LEP,TGFB1,TNF,EPAS1
Signaling by EGFR	1.86E-9	4.31E-8	18	CDKN1B,ERBB2,PAG1,PIK3CA,FGFR1,ADAM17,FGFR2,KIT,KRAS,RAF1,HBEGF,GSK3A,GSK3B,EGFR,PRKCA,PRKCE,MAPK1,MAPK3
a6b1 and a6b4 Integrin signaling	2.02E-9	4.52E-8	9	ERBB2,ITGA6,ITGB1,ITGB4,MET,PIK3CA,YWHAZ,EGFR,PRKCA
EGF Signaling Pathway	2.02E-9	4.52E-8	9	SRF,STAT3,STAT5A,STAT5B,PIK3CA,RAF1,EGFR,PRKCA,MAPK3
Non-integrin membrane-ECM interactions	2.13E-9	4.64E-8	10	ITGA6,ITGB1,ITGB3,ITGB4,SDC1,VTN,DDR2,LAMA5,TGFB1,PRKCA
Signaling by Leptin	2.14E-9	4.64E-8	8	STAT3,STAT5A,STAT5B,KRAS,RAF1,LEP,MAPK1,MAPK3
Signaling by EGFR in Cancer	2.24E-9	4.77E-8	18	CDKN1B,ERBB2,PAG1,PIK3CA,FGFR1,ADAM17,FGFR2,KIT,KRAS,RAF1,HBEGF,GSK3A,GSK3B,EGFR,PRKCA,PRKCE,MAPK1,MAPK3
Wnt Signaling Pathway and Pluripotency	2.43E-9	4.93E-8	14	MYC,AXIN1,AXIN2,WNT3A,MMP7,WNT1,POU5F1,GSK3B,FOSL1,CD44,LRP6,PRKCA,PRKCE,CTNND1
Neural Crest Differentiation	2.43E-9	4.93E-8	14	MYC,SOX9,ITGB1,AXIN1,AXIN2,TWIST1,BMP4,WNT3A,FGFR1,FGFR2,NOTCH2,WNT1,GSK3B,SNAI2
Transcriptional misregulation in cancer	2.45E-9	4.93E-8	18	MYC,CDKN1B,MYCN,SP1,ETV4,PTK2,DDX5,HOXA10,BMI1,MET,MMP3,FLT1,ZEB1,WT1,ID2,PPARG,IGF1R,IGFBP3
Signaling by FGFR	2.46E-9	4.93E-8	17	CDKN1B,ERBB2,BRAF,PIK3CA,FGFR1,FGFR2,KIT,KRAS,RAF1,HBEGF,GSK3A,GSK3B,EGFR,PRKCA,PRKCE,MAPK1,MAPK3
DAP12 signaling	2.71E-9	5.32E-8	17	CDKN1B,ERBB2,PIK3CA,FGFR1,FGFR2,KIT,RAC1,KRAS,RAF1,HBEGF,GSK3A,GSK3B,EGFR,PRKCA,PRKCE,MAPK1,MAPK3
ErbB4 signaling events	2.77E-9	5.32E-8	9	ERBB2,STAT5A,STAT5B,PIK3CA,YAP1,ADAM17,HBEGF,MAPK1,MAPK3
Osteopontin-mediated events	2.77E-9	5.32E-8	9	ITGB3,PIK3CA,RAC1,ROCK2,GSN,CD44,PAK1,MAPK1,MAPK3
ErbB1 downstream signaling	3.15E-9	5.97E-8	14	MYC,SRF,STAT3,BRAF,PIK3CA,RAC1,RAF1,YWHAZ,EGFR,EGR1,PRKCA,MAPK1,MAPK3,MAPK7
MAPKinase Signaling Pathway	3.59E-9	6.65E-8	13	MYC,SP1,MAP3K3,BRAF,RAC1,RAF1,TGFB1,TGFB2,MAPK14,PAK1,MAPK1,MAPK3,MAPK7

Acute myeloid leukemia	3.61E-9	6.65E-8	11	MYC,STAT3,STAT5A,STAT5B,BRAF,PIK3CA,KIT,KRAS,RAF1,MAPK1,MAPK3
Signaling of Hepatocyte Growth Factor Receptor	3.76E-9	6.82E-8	9	HGF,ITGB1,PTK2,STAT3,PIK3CA,RAF1,PAK1,MAPK1,MAPK3
VEGF signaling pathway	4.39E-9	7.87E-8	11	PTK2,BRAF,PIK3CA,VEGFA,RAF1,TGFB1I1,MAPK14,PRKCA,PRKCE,MAPK1,MAPK3
Downstream signaling of activated FGFR	4.63E-9	8.20E-8	16	CDKN1B,ERBB2,PIK3CA,FGFR1,FGFR2,KIT,KRAS,RAF1,HBEGF,GSK3A,GSK3B,EGFR,PRKCA,PRKCE,MAPK1,MAPK3
Erk and PI-3 Kinase Are Necessary for Collagen Binding in Corneal Epithelia	4.78E-9	8.35E-8	8	ITGB1,PTK2,PIK3CA,RAF1,GSN,ROCK1,MAPK1,MAPK3
Developmental Biology	4.97E-9	8.58E-8	27	ERBB2,PLXND1,ITGA5,ITGB1,ITGB3,PTK2,MET,FGFR1,RAC1,ROCK2,KRAS,RAF1,WNT1,PPARG,LEP,GSK3B,TGFB1,LEFTY1,MAPK14,EGFR,CSNK2B,PAK1,ROCK1,TNF,MAPK1,MAPK3,SEMA3E
MAPK signaling pathway	5.27E-9	8.97E-8	17	MYC,SRF,BRAF,HSPB1,RAC1,KRAS,RAF1,TGFB1,TGFB2,MAPK14,EGFR,PAK1,TNF,MAPK1,MAPK3,MAPK7,IL1B
MAPK signaling pathway	5.52E-9	9.28E-8	21	MYC,SRF,MAP3K3,BRAF,FGFR1,FGFR2,HSPB1,RAC1,KRAS,RAF1,TGFB1,TGFB2,MAPK14,EGFR,PAK1,PRKCA,TNF,MAPK1,MAPK3,MAPK7,IL1B
Integrin signalling pathway	5.77E-9	9.60E-8	17	ITGA6,ITGA5,ITGB1,ITGB3,ITGB4,PTK2,MAP3K3,BRAF,PIK3CA,RAC1,RAF1,COL8A1,COL8A2,LAMA5,CAV1,LIMS1,MAPK3
Leptin signaling pathway	6.40E-9	1.05E-7	11	SP1,ERBB2,STAT3,STAT5B,RAC1,RAF1,LEP,MAPK14,KHDRBS1,MAPK1,MAPK3
FGF signaling pathway	6.95E-9	1.13E-7	10	HGF,STAT5B,MET,PIK3CA,PLAUR,FGFR1,FGFR2,MAPK1,MAPK3,CTNND1
Interleukin signaling pathway	7.24E-9	1.16E-7	13	MYC,CDKN1B,SRF,STAT3,STAT5A,STAT5B,BRAF,PIK3CA,RAF1,GSK3B,MAPK1,MAPK3,MAPK7
Signaling events mediated by TCPTP	8.76E-9	1.39E-7	9	HGF,ITGB1,STAT3,STAT5A,STAT5B,MET,PIK3CA,VEGFA,EGFR
The extracellular signal-regulated RAF/MEK/ERK signaling	9.85E-9	1.54E-7	8	MDK,BRAF,KRAS,RAF1,FLT1,EGFR,MAPK1,MAPK3
TSH signaling pathway	1.31E-8	2.02E-7	11	MYC,CDKN1B,STAT3,BRAF,PIK3CA,RAF1,MAPK14,IGF1R,SCRIB,MAPK1,MAPK3
Bladder cancer	1.47E-8	2.23E-7	9	MYC,ERBB2,BRAF,VEGFA,KRAS,RAF1,EGFR,MAPK1,MAPK3
TSLP Signaling Pathway	1.47E-8	2.23E-7	9	MYC,STAT3,GAB2,STAT5A,STAT5B,PIK3CA,MAPK14,MAPK1,MAPK3
DAP12 interactions	1.66E-8	2.48E-7	17	CDKN1B,ERBB2,PIK3CA,FGFR1,FGFR2,KIT,RAC1,KRAS,RAF1,HBEGF,GSK3A,GSK3B,EGFR,PRKCA,PRKCE,MAPK1,MAPK3
Integrin Signaling Pathway	1.86E-8	2.76E-7	12	ITGA6,ITGA5,ZYX,PTK2,BRAF,PIK3CA,ROCK2,RAF1,CAV1,PAK1,ROCK1,MAPK1
Growth Hormone Signaling Pathway	1.90E-8	2.76E-7	8	SRF,STAT5A,STAT5B,PIK3CA,RAF1,PRKCA,MAPK1,MAPK3

Erk1/Erk2 Mapk Signaling pathway	1.90E-8	2.76E-7	8	MYC,ITGB1,STAT3,RAF1,IGF1R,EGFR,MAPK1,MAPK3
Signaling by ERBB2	1.95E-8	2.80E-7	16	CDKN1B,ERBB2,PIK3CA,FGFR1,FGFR2,KIT,KRAS,RAF1,HBEGF,GSK3A,GSK3B,EGFR,PRKCA,PRKCE,MAPK1,MAPK3
Endothelins	2.39E-8	3.39E-7	10	RAC1,RAF1,EDN1,EDNRA,MAPK14,PAK1,PRKCA,PRKCE,MAPK1,MAPK3
Trk receptor signaling mediated by the MAPK pathway	2.59E-8	3.63E-7	8	SRF,BRAF,RAF1,MAPK14,EGR1,MAPK1,MAPK3,MAPK7
Signaling events mediated by focal adhesion kinase	2.89E-8	4.01E-7	10	ITGA5,ITGB1,BRAF,PIK3CA,MMP14,RAC1,ROCK2,RAF1,PAK1,MAPK1
Elastic fibre formation	3.02E-8	4.16E-7	9	ITGA5,ITGB1,ITGB3,BMP2,BMP4,LOXL3,VTN,TGFB1,TGFB2
Fc epsilon RI signaling pathway	3.47E-8	4.72E-7	11	GAB2,PIK3CA,RAC1,KRAS,RAF1,MAPK14,PRKCA,PRKCE,TNF,MAPK1,MAPK3
Axon guidance	3.62E-8	4.88E-7	20	ERBB2,PLXND1,ITGA5,ITGB1,ITGB3,PTK2,MET,FGFR1,RAC1,ROCK2,KRAS,RAF1,GSK3B,EGFR,CSNK2B,PAK1,ROCK1,MAPK1,MAPK3,SEMA3E
Chemokine signaling pathway	3.73E-8	4.98E-7	17	PTK2,STAT3,STAT5B,BRAF,PIK3CA,CXCR4,CXCL5,RAC1,ROCK2,KRAS,RAF1,GSK3A,GSK3B,PAK1,ROCK1,MAPK1,MAPK3
Melanoma	4.04E-8	5.34E-7	11	HGF,MET,BRAF,PIK3CA,FGFR1,KRAS,RAF1,IGF1R,EGFR,MAPK1,MAPK3
HIF-1 signaling pathway	4.16E-8	5.37E-7	13	CDKN1B,EPO,ERBB2,STAT3,PIK3CA,VEGFA,FLT1,EDN1,IGF1R,EGFR,PRKCA,MAPK1,MAPK3
Non-small cell lung cancer	4.16E-8	5.37E-7	10	ERBB2,BRAF,PIK3CA,KRAS,RAF1,TGFA,EGFR,PRKCA,MAPK1,MAPK3
Hepatitis B	4.17E-8	5.37E-7	15	MYC,CDKN1B,STAT3,STAT5A,STAT5B,PIK3CA,KRAS,RAF1,YWHAZ,TGFB1,TGFB2,PRKCA,TNF,MAPK1,MAPK3
Androgen receptor signaling pathway	4.21E-8	5.37E-7	12	SP1,PTK2,STAT3,FHL2,RAC1,ROCK2,CAV1,GSK3B,TGFB1I1,EGFR,PRDX1,ROCK1
Regulation of Actin Cytoskeleton	4.58E-8	5.77E-7	15	PTK2,BRAF,PIK3CA,FGFR1,FGFR2,RAC1,ROCK2,KRAS,RAF1,GSN,EGFR,PAK1,ROCK1,MAPK1,MAPK3
Prolactin signaling pathway	4.69E-8	5.84E-7	11	STAT3,STAT5A,STAT5B,PIK3CA,KRAS,RAF1,GSK3B,MAPK14,ELF5,MAPK1,MAPK3
TWEAK Signaling Pathway	4.71E-8	5.84E-7	9	RAC1,RAF1,SHH,BIRC2,GSK3B,MAPK14,TNF,MAPK1,MAPK3
Axon guidance	4.90E-8	6.02E-7	14	ITGB1,PTK2,SEMA4C,MET,CXCR4,RAC1,ROCK2,KRAS,GSK3B,PAK1,ROCK1,MAPK1,MAPK3,SEMA3E
Adherens junction	5.44E-8	6.56E-7	11	ERBB2,MET,FGFR1,RAC1,IGF1R,EGFR,CSNK2B,SNAI2,MAPK1,MAPK3,CTNND1
Corticotropin-releasing hormone	5.45E-8	6.56E-7	12	SP1,PTK2,PARP1,BRAF,RAF1,GSK3B,FOSL1,TGFB1,MAPK14,PRKCA,MAPK1,MAPK3
Fc epsilon receptor (FCERI) signaling	5.49E-8	6.56E-7	17	CDKN1B,ERBB2,GAB2,PIK3CA,FGFR1,FGFR2,KIT,RAC1,KRAS,RAF1,HBEGF,GSK3A,GSK3B,EGFR,PAK1,MAPK1,MAPK3
Prostate Cancer	5.84E-8	6.92E-7	13	MYC,HGF,SP1,ETV4,FOXA1,STAT3,NDRG1,PIK3CA,RAF1,MAPK14,PAK1,MAPK1,MAPK3

Mechanism of Gene Regulation by Peroxisome Proliferators via PPARa(alpha)	5.92E-8	6.95E-7	10	MYC,SP1,PTGS2,STAT5A,STAT5B,PIK3CA,PRKCA,TNF,MAPK1,MAPK3
PDGF Signaling Pathway	6.06E-8	7.06E-7	8	SRF,STAT3,STAT5A,STAT5B,PIK3CA,RAF1,PRKCA,MAPK3
TGF-beta signaling pathway	6.18E-8	7.08E-7	12	ACVR1,BMP2,BMP4,ZFYVE9,AMHR2,FOSL1,TGFB2,LEFTY1,MAPK14,MAPK1,MAPK3,SMAD9
DNA damage response (only ATM dependent)	6.18E-8	7.08E-7	12	MYC,CDKN1B,ERBB2,HMGB1,AXIN1,WNT3A,RAC1,WNT1,GSK3B,FOSL1,TGFB1,MAPK1
TNF signaling pathway	6.52E-8	7.40E-7	13	PTGS2,PIK3CA,JAG1,MMP3,MMP14,CXCL5,BIRC2,EDN1,MAPK14,TNF,MAPK1,MAPK3,IL1B
Signaling by FGFR mutants	7.18E-8	8.09E-7	9	STAT3,GAB2,STAT5A,STAT5B,PIK3CA,FGFR1,FGFR2,KRAS,CUX1
Osteoclast differentiation	8.02E-8	8.96E-7	14	ITGB3,GAB2,PIK3CA,FHL2,RAC1,PPARG,FOSL1,TGFB1,TGFB2,MAPK14,TNF,MAPK1,MAPK3,IL1B
Keratinocyte Differentiation	8.80E-8	9.75E-7	9	SP1,RAF1,MAPK14,EGFR,PRKCA,PRKCE,TNF,MAPK1,MAPK3
GRB2 events in ERBB2 signaling	9.37E-8	1.03E-6	7	ERBB2,KRAS,RAF1,HBEGF,EGFR,MAPK1,MAPK3
Angiotensin II mediated activation of JNK Pathway via Pyk2 dependent signaling	1.01E-7	1.10E-6	8	PTK2,RAC1,RAF1,EGFR,PAK1,PRKCA,MAPK1,MAPK3
HIF-2-alpha transcription factor network	1.01E-7	1.10E-6	8	EPO,SP1,TWIST1,MMP14,VEGFA,FLT1,POU5F1,EPAS1
Ras signaling pathway	1.02E-7	1.10E-6	18	HGF,GAB2,MET,PIK3CA,FGFR1,FGFR2,KIT,RAC1,VEGFA,KRAS,RAF1,FLT1,IGF1R,EGFR,PAK1,PRKCA,MAPK1,MAPK3
Heart Development	1.07E-7	1.14E-6	9	SRF,BMP2,BMP4,TBX20,FOXC1,TBX2,VEGFA,SHH,MAPK1
Signaling by Interleukins	1.23E-7	1.30E-6	13	HGF,STAT3,GAB2,MAP3K3,STAT5A,STAT5B,PIK3CA,KRAS,RAF1,YWHAZ,MAPK1,MAPK3,IL1B
CXCR4 Signaling Pathway	1.30E-7	1.35E-6	7	PTK2,PIK3CA,CXCR4,RAF1,PRKCA,MAPK1,MAPK3
ALK1 signaling events	1.30E-7	1.35E-6	7	ACVR1,CAV1,TGFB1,CSNK2B,MAPK1,MAPK3,SMAD9
TPO Signaling Pathway	1.30E-7	1.35E-6	7	STAT3,STAT5A,STAT5B,PIK3CA,RAF1,PRKCA,MAPK3
Agrin in Postsynaptic Differentiation	1.64E-7	1.68E-6	8	SP1,ITGB1,PTK2,RAC1,EGFR,PAK1,MAPK1,MAPK3
EGF receptor (ErbB1) signaling pathway	1.78E-7	1.80E-6	7	STAT3,PIK3CA,GSN,EGFR,PAK1,MAPK1,MAPK3
SHC1 events in ERBB2 signaling	1.78E-7	1.80E-6	7	ERBB2,KRAS,RAF1,HBEGF,EGFR,MAPK1,MAPK3
Glioma	1.82E-7	1.83E-6	10	BRAF,PIK3CA,KRAS,RAF1,TGFA,IGF1R,EGFR,PRKCA,MAPK1,MAPK3

Downstream signaling in naive CD8+ T cells	1.88E-7	1.87E-6	9	BRAF,RAF1,FOSL1,EGR1,PRKCA,PRKCE,TNF,MAPK1,MAPK3
EGFR Transactivation by Gastrin	1.96E-7	1.92E-6	5	MMP3,KRAS,HBEGF,EGFR,PRKCA
Cadmium induces DNA synthesis and proliferation in macrophages	1.96E-7	1.92E-6	6	MYC,RAF1,PRKCA,TNF,MAPK1,MAPK3
Endochondral Ossification	2.12E-7	2.06E-6	10	SOX9,PTHLH,STAT5B,FGFR1,MMP13,VEGFA,DDR2,TGFB1,TGFB2,IGF1R
FGF signaling pathway	2.23E-7	2.16E-6	12	MAP3K3,PIK3CA,FGFR1,FGFR2,RAC1,RAF1,YWHAZ,MAPK14,PRKCA,PRKCE,MAPK1,MAPK3
BDNF signaling pathway	2.40E-7	2.28E-6	14	STAT3,STAT5A,STAT5B,BMP2,ADAM17,RAC1,RAF1,YBX1,GSK3B,MAPK14,EGR1,MAPK1,MAPK3,MAPK7
EPO Receptor Signaling	2.40E-7	2.28E-6	7	EPO,STAT3,STAT5A,STAT5B,RAF1,MAPK1,MAPK3
Influence of Ras and Rho proteins on G1 to S Transition	2.40E-7	2.28E-6	7	CDKN1B,PIK3CA,RAC1,RAF1,PAK1,MAPK1,MAPK3
Integrin Signaling Pathway	2.57E-7	2.42E-6	8	ITGB1,ZYX,PTK2,RAF1,CAV1,ROCK1,MAPK1,MAPK3
Signaling events mediated by Stem cell factor receptor (c-Kit)	2.68E-7	2.49E-6	9	EPO,STAT3,STAT5A,PIK3CA,KIT,RAF1,GSK3B,SNAI2,MAPK3
Signaling events mediated by PTP1B	2.68E-7	2.49E-6	9	ITGB3,STAT3,STAT5A,STAT5B,PIK3CA,YBX1,CAV1,LEP,EGFR
Semaphorin interactions	2.83E-7	2.61E-6	10	ERBB2,PLXND1,ITGB1,MET,RAC1,ROCK2,GSK3B,PAK1,ROCK1,SEMA3E
Phospholipids as signalling intermediaries	3.20E-7	2.93E-6	7	ITGB3,PTK2,PIK3CA,RAC1,PRKCA,MAPK1,MAPK3
Stabilization and expansion of the E-cadherin adherens junction	3.92E-7	3.57E-6	8	HGF,ZYX,MET,LIMA1,IGF1R,EGFR,ROCK1,CTNND1
S1P3 pathway	4.20E-7	3.79E-6	7	ITGB3,CXCR4,RAC1,VEGFA,FLT1,MAPK1,MAPK3
CDC42 signaling events	4.29E-7	3.85E-6	10	BRAF,PIK3CA,RAC1,RAF1,GSK3B,MAPK14,PAK1,PRKCE,MAPK1,MAPK3
PTEN dependent cell cycle arrest and apoptosis	4.41E-7	3.93E-6	6	CDKN1B,ITGB1,PTK2,PIK3CA,MAPK1,MAPK3
Signaling events mediated by Hepatocyte Growth Factor Receptor (c-Met)	4.91E-7	4.34E-6	10	HGF,GAB2,MET,PIK3CA,RAC1,RAF1,EGR1,PAK1,MAPK1,MAPK3
VEGFR1 specific signals	5.46E-7	4.74E-6	7	PIK3CA,VEGFA,FLT1,CAV1,PRKCA,MAPK1,MAPK3
MAPK Cascade	5.46E-7	4.74E-6	7	MAP3K3,BRAF,KRAS,RAF1,MAPK14,MAPK1,MAPK3
Links between Pyk2 and Map Kinases	5.46E-7	4.74E-6	7	RAC1,RAF1,MAPK14,PAK1,PRKCA,MAPK1,MAPK3

IL-2 Signaling pathway	5.84E-7	5.04E-6	8	MYC,STAT3,GAB2,STAT5A,STAT5B,RAF1,MAPK1,MAPK3
GnRH signaling pathway	6.14E-7	5.24E-6	11	MAP3K3,MMP14,KRAS,RAF1,HBEGF,MAPK14,EGFR,PRKCA,MAPK1,MAPK3,MAPK7
Hepatitis C	6.15E-7	5.24E-6	13	STAT3,BRAF,PIK3CA,KRAS,RAF1,CLDN4,GSK3B,CLDN1,MAPK14,EGFR,TNF,MAPK1,MAPK3
Signaling by FGFR1 fusion mutants	6.35E-7	5.34E-6	6	STAT3,GAB2,STAT5A,STAT5B,PIK3CA,CUX1
Role of MAL in Rho-Mediated Activation of SRF	6.35E-7	5.34E-6	6	SRF,RAC1,RAF1,ROCK1,MAPK1,MAPK3
Direct p53 effectors	6.71E-7	5.61E-6	13	HGF,SP1,FOXA1,DDX5,MET,NDRG1,VDR,CAV1,TGFA,IGFBP3,EGFR,TP63,SNAI2
Ras signaling in the CD4+ TCR pathway	6.97E-7	5.74E-6	5	BRAF,RAF1,PRKCA,MAPK1,MAPK3
Molecules associated with elastic fibres	7.01E-7	5.74E-6	7	ITGB1,ITGB3,BMP2,BMP4,VTN,TGFB1,TGFB2
Signaling by FGFR1 mutants	7.01E-7	5.74E-6	7	STAT3,GAB2,STAT5A,STAT5B,PIK3CA,FGFR1,CUX1
Bioactive Peptide Induced Signaling Pathway	7.07E-7	5.74E-6	8	STAT3,STAT5A,STAT5B,RAF1,MAPK14,PRKCA,MAPK1,MAPK3
Interleukin-2 signaling	7.07E-7	5.74E-6	8	GAB2,STAT5A,STAT5B,PIK3CA,KRAS,RAF1,MAPK1,MAPK3
CXCR4-mediated signaling events	8.22E-7	6.63E-6	10	PAG1,ITGA6,ITGB1,PTK2,STAT3,STAT5A,STAT5B,PIK3CA,CXCR4,PAK1
Regulation of Microtubule Cytoskeleton	8.51E-7	6.82E-6	8	STAT3,PIK3CA,WNT3A,RAC1,GSK3B,PAK1,PRKCA,ROCK1
Syndecan-4-mediated signaling events	8.92E-7	7.08E-6	7	ITGA5,MDK,ITGB1,CXCR4,FGFR1,RAC1,PRKCA
S1P1 pathway	8.94E-7	7.08E-6	6	ITGB3,PTGS2,RAC1,VEGFA,MAPK1,MAPK3
Wnt Signaling Pathway	9.54E-7	7.51E-6	9	MYC,AXIN1,WNT3A,RAC1,WNT1,GSK3B,FOSL1,PRKCA,PRKCE
Wnt signaling pathway	1.02E-6	8.01E-6	13	MYC,AXIN1,AXIN2,WNT3A,MMP7,RAC1,ROCK2,WNT1,GSK3B,FOSL1,CSNK2B,LRP6,PRKCA
Signal Transduction	1.05E-6	8.15E-6	61	MYC,CDKN1B,SP1,ERBB2,PAG1,PDE4A,NOG,MDK,ITGB3,PTHLH,PTK2,STAT3,GAB2,STAT5A,AXIN1,STAT5B,BMP2,PARP1,ZFYVE9,BRAF,PIK3CA,CXCR4,YAP1,WNT3A,JAG1,FGFR1,ADAM17,FGFR2,MMP3,CXCL5,KIT,SDC1,NOTCH2,RAC1,VEGFA,ROCK2,KRAS,RAF1,FLT1,ANXA1,HBEGF,WNT1,SHH,WWTR1,ECT2,LEP,EDN1,GSK3A,GSK3B,EDNRA,TGFB1,MAPK14,IGF1R,EGFR,PRKCA,PRKCE,ROCK1,MAPK1,MAPK3,MAPK7,SMAD9
Insulin Signaling	1.10E-6	8.44E-6	14	SRF,MAP3K3,PIK3CA,RAC1,RAF1,GSK3A,GSK3B,MAPK14,IGF1R,EGR1,PRKCA,MAPK1,MAPK3,MAPK7
Shigellosis	1.10E-6	8.44E-6	9	ITGA5,ITGB1,RAC1,ROCK2,MAPK14,CD44,ROCK1,MAPK1,MAPK3

Leukocyte transendothelial migration	1.10E-6	8.44E-6	12	ITGB1,PTK2,PIK3CA,CXCR4,RAC1,ROCK2,CLDN4,CLDN1,MAPK14,PRKCA,ROCK1,CTNND1
Trefoil Factors Initiate Mucosal Healing	1.23E-6	9.39E-6	6	ITGB1,PTK2,PIK3CA,EGFR,MAPK1,MAPK3
Validated targets of C-MYC transcriptional repression	1.27E-6	9.61E-6	9	MYC,CDKN1B,SP1,ERBB2,ITGA6,ITGB1,ITGB4,NDRG1,ID2
IL-6 Signaling Pathway	1.29E-6	9.74E-6	11	ERBB2,PTK2,STAT3,GAB2,HSPB1,RAC1,RAF1,GSK3B,MAPK14,MAPK1,MAPK3
N-cadherin signaling events	1.41E-6	1.05E-5	7	AXIN1,PIK3CA,FGFR1,RAC1,GSN,ROCK1,CTNND1
Toxoplasmosis	1.58E-6	1.18E-5	12	ITGA6,ITGB1,STAT3,PIK3CA,LAMA5,BIRC2,TGFB1,TGFB2,MAPK14,TNF,MAPK1,MAPK3
Aspirin Blocks Signaling Pathway Involved in Platelet Activation	1.67E-6	1.23E-5	6	ITGB1,PTK2,RAF1,PRKCA,MAPK1,MAPK3
Role of ERBB2 in Signal Transduction and Oncology	1.67E-6	1.23E-5	6	STAT3,PIK3CA,RAF1,EGFR,MAPK1,MAPK3
HIF-1-alpha transcription factor network	1.91E-6	1.40E-5	9	EPO,SP1,NDRG1,CXCR4,VEGFA,ID2,LEP,EDN1,ENG
GAB1 signalosome	1.92E-6	1.40E-5	11	CDKN1B,ERBB2,PAG1,PIK3CA,FGFR1,FGFR2,KIT,HBEGF,GSK3A,GSK3B,EGFR
CTCF: First Multivalent Nuclear Factor	2.23E-6	1.57E-5	6	MYC,CDKN1B,PIK3CA,TGFB1,TGFB2,TGFBR3
CCR3 signaling in Eosinophils	2.23E-6	1.57E-5	6	PTK2,ROCK2,RAF1,PRKCA,MAPK1,MAPK3
Inhibition of Cellular Proliferation by Gleevec	2.23E-6	1.57E-5	6	MYC,STAT5A,STAT5B,PIK3CA,RAF1,MAPK3
Angiogenesis	2.23E-6	1.57E-5	6	PTK2,PIK3CA,FGFR2,VEGFA,FLT1,MAPK1
VEGFR3 signaling in lymphatic endothelium	2.23E-6	1.57E-5	6	ITGA5,ITGB1,PIK3CA,MAPK14,MAPK1,MAPK3
Signaling events mediated by PRL	2.23E-6	1.57E-5	6	ITGB1,RAC1,EGR1,ROCK1,MAPK1,MAPK3
Genes related to PIP3 signaling in cardiac myocytes	2.48E-6	1.74E-5	9	MYC,CDKN1B,PTK2,MET,PIK3CA,YWHAZ,GSK3A,GSK3B,PAK1
GRB2 events in EGFR signaling	2.89E-6	2.02E-5	5	KRAS,RAF1,EGFR,MAPK1,MAPK3
Constitutive PI3K/AKT Signaling in Cancer	2.91E-6	2.02E-5	10	CDKN1B,ERBB2,PIK3CA,FGFR1,FGFR2,KIT,HBEGF,GSK3A,GSK3B,EGFR

Physiological and Pathological Hypertrophy of the Heart	2.93E-6	2.02E-5	6	STAT3,RAC1,EDN1,MAPK14,PRKCE,MAPK1
Growth hormone receptor signaling	2.93E-6	2.02E-5	6	STAT3,STAT5A,STAT5B,ADAM17,MAPK1,MAPK3
Hematopoietic cell lineage	3.23E-6	2.22E-5	10	EPO,ITGA6,ITGA5,ITGB3,KIT,ANPEP,CD44,CD24,TNF,IL1B
Cell-Cell communication	3.36E-6	2.29E-5	12	ITGA6,ITGB1,ITGB4,PTK2,PIK3CA,RAC1,CLDN4,CLDN1,LIMS1,MAPK14,PAK1,CTNND1
integrin signaling	3.86E-6	2.60E-5	7	ITGA6,ITGA5,ITGB1,ITGB3,ITGB4,PTK2,LIMS1
IL-2 Receptor Beta Chain in T cell Activation	3.86E-6	2.60E-5	7	MYC,STAT5A,STAT5B,PIK3CA,RAF1,MAPK1,MAPK3
Cytokines can induce activation of matrix metalloproteinases, which degrade extracellular matrix.	4.28E-6	2.83E-5	5	TGFB1,TGFB2,CD44,TNF,IL1B
Signalling to p38 via RIT and RIN	4.28E-6	2.83E-5	5	BRAF,KRAS,RAF1,MAPK1,MAPK3
SHC1 events in EGFR signaling	4.28E-6	2.83E-5	5	KRAS,RAF1,EGFR,MAPK1,MAPK3
Assembly of collagen fibrils and other multimeric structures	4.30E-6	2.83E-5	8	ITGA6,ITGB4,LOXL3,MMP3,MMP7,MMP13,COL8A1,COL8A2
NFAT and Hypertrophy of the heart (Transcription in the broken heart)	4.30E-6	2.83E-5	8	PIK3CA,RAF1,HBEGF,EDN1,GSK3B,MAPK14,MAPK1,MAPK3
TCR Signaling Pathway	4.40E-6	2.87E-5	10	GATA3,GAB2,RAF1,VIM,TGFB1,MAPK14,PAK1,MAPK1,MAPK3,IL1B
Fc gamma R-mediated phagocytosis	4.40E-6	2.87E-5	10	GAB2,PIK3CA,RAC1,RAF1,GSN,PAK1,PRKCA,PRKCE,MAPK1,MAPK3
Cytokine-cytokine receptor interaction	4.49E-6	2.91E-5	17	EPO,HGF,ACVR1,MET,BMP2,CXCR4,CXCL5,KIT,VEGFA,AMHR2,FLT1,LEP,TGFB1,TGFB2,EGFR,TNF,IL1B
IL27-mediated signaling events	4.86E-6	3.14E-5	6	GATA3,STAT3,STAT5A,TGFB1,TNF,IL1B
Basal cell carcinoma	4.95E-6	3.18E-5	8	AXIN1,AXIN2,BMP2,BMP4,WNT3A,WNT1,SHH,GSK3B
Endothelin signaling pathway	5.14E-6	3.29E-5	9	PTGS2,PIK3CA,RAF1,EDN1,EDNRA,PRKCA,PRKCE,MAPK1,MAPK3
Platelet activation, signaling and aggregation	5.76E-6	3.65E-5	15	HGF,ITGB3,PTK2,PIK3CA,RAC1,VEGFA,RAF1,YWHAZ,TGFB1,TGFB2,MAPK14,PRKCA,PRKCE,MAPK1,MAPK3
L1CAM interactions	5.91E-6	3.73E-5	10	ITGA5,ITGB1,ITGB3,FGFR1,RAC1,EGFR,CSNK2B,PAK1,MAPK1,MAPK3
ARMS-mediated activation	6.13E-6	3.83E-5	5	BRAF,KRAS,RAF1,MAPK1,MAPK3

Genes related to IL4 rceptor signaling in B lymphocytes	6.16E-6	3.83E-5	6	PIK3CA,RAF1,GSK3A,GSK3B,MAPK1,MAPK3
Transcription factor CREB and its extracellular signals	6.16E-6	3.83E-5	6	PIK3CA,RAC1,MAPK14,PRKCA,MAPK1,MAPK3
Leishmaniasis	7.20E-6	4.44E-5	9	ITGB1,PTGS2,TGFB1,TGFB2,MAPK14,TNF,MAPK1,MAPK3,IL1B
Human Cytomegalovirus and Map Kinase Pathways	8.57E-6	5.24E-5	5	SP1,PIK3CA,MAPK14,MAPK1,MAPK3
Tumor Suppressor Arf Inhibits Ribosomal Biogenesis	8.57E-6	5.24E-5	5	MYC,TWIST1,PIK3CA,RAC1,TBX2
Neurotrophin signaling pathway	8.62E-6	5.25E-5	11	MAP3K3,BRAF,PIK3CA,RAC1,KRAS,RAF1,GSK3B,MAPK14,MAPK1,MAPK3,MAPK7
PI3K events in ERBB4 signaling	9.43E-6	5.62E-5	10	CDKN1B,ERBB2,PIK3CA,FGFR1,FGFR2,KIT,HBEGF,GSK3A,GSK3B,EGFR
PI3K/AKT Signaling in Cancer	9.43E-6	5.62E-5	10	CDKN1B,ERBB2,PIK3CA,FGFR1,FGFR2,KIT,HBEGF,GSK3A,GSK3B,EGFR
PIP3 activates AKT signaling	9.43E-6	5.62E-5	10	CDKN1B,ERBB2,PIK3CA,FGFR1,FGFR2,KIT,HBEGF,GSK3A,GSK3B,EGFR
PI-3K cascade	9.43E-6	5.62E-5	10	CDKN1B,ERBB2,PIK3CA,FGFR1,FGFR2,KIT,HBEGF,GSK3A,GSK3B,EGFR
PI3K events in ERBB2 signaling	9.43E-6	5.62E-5	10	CDKN1B,ERBB2,PIK3CA,FGFR1,FGFR2,KIT,HBEGF,GSK3A,GSK3B,EGFR
Thyroid cancer	9.60E-6	5.69E-5	6	MYC,BRAF,KRAS,PPARG,MAPK1,MAPK3
IL-7 Signaling Pathway	1.07E-5	6.31E-5	7	STAT3,STAT5A,STAT5B,RAF1,GSK3B,MAPK1,MAPK3
Melanogenesis	1.13E-5	6.63E-5	10	WNT3A,KIT,KRAS,RAF1,WNT1,EDN1,GSK3B,PRKCA,MAPK1,MAPK3
colorectal cancer	1.17E-5	6.71E-5	5	AXIN1,AXIN2,BRAF,KRAS,TGFB1
Role of EGF Receptor Transactivation by GPCRs in Cardiac Hypertrophy	1.17E-5	6.71E-5	5	MYC,EDN1,EDNRA,EGFR,PRKCA
Frs2-mediated activation	1.17E-5	6.71E-5	5	BRAF,KRAS,RAF1,MAPK1,MAPK3
Telomeres, Telomerase, Cellular Aging, and Immortality	1.17E-5	6.71E-5	5	MYC,KRAS,IGF1R,EGFR,PRKCA
SHC-related events triggered by IGF1R	1.17E-5	6.71E-5	5	KRAS,RAF1,IGF1R,MAPK1,MAPK3
Role of LAT2/NTAL/LAB on calcium mobilization	1.18E-5	6.71E-5	11	CDKN1B,ERBB2,GAB2,PIK3CA,FGFR1,FGFR2,KIT,HBEGF,GSK3A,GSK3B,EGFR
Canonical Wnt signaling pathway	1.18E-5	6.71E-5	6	AXIN1,AXIN2,WNT3A,CAV1,GSK3B,LRP6
IL2 signaling events mediated by STAT5	1.18E-5	6.71E-5	6	MYC,SP1,GAB2,STAT5A,STAT5B,PIK3CA

PI3K/AKT activation	1.23E-5	6.97E-5	10	CDKN1B,ERBB2,PIK3CA,FGFR1,FGFR2,KIT,HBEGF,GSK3A,GSK3B,EGFR
Presenilin action in Notch and Wnt signaling	1.45E-5	8.17E-5	7	MYC,AXIN1,WNT1,GSK3B,LRP6,MAPK1,MAPK3
Cytokine Signaling in Immune system	1.55E-5	8.67E-5	17	HGF,STAT3,GAB2,MAP3K3,STAT5A,STAT5B,PIK3CA,PIN1,ADAM17,KRAS,RAF1,YWHAZ,EGR1,CD44,MAPK1,MAPK3,IL1B
EPO Signaling Pathway	1.56E-5	8.67E-5	5	EPO,STAT5A,STAT5B,RAF1,MAPK3
E-cadherin signaling in keratinocytes	1.56E-5	8.67E-5	5	ZYX,PIK3CA,RAC1,EGFR,CTNND1
Serotonin Receptor 4/6/7 and NR3C Signaling	1.56E-5	8.67E-5	5	SRF,BRAF,EGR1,MAPK1,MAPK3
amb2 Integrin signaling	1.94E-5	1.06E-4	7	HMGB1,AGER,PLAUR,VTN,CYR61,ROCK1,TNF
Hemostasis	1.94E-5	1.06E-4	23	HGF,GATA3,ITGA6,ITGA5,ITGB1,ITGB3,PTK2,PIK3CA,PLAUR,RAC1,VEGFA,KRAS,RAF1,CAV1,YWHAZ,TGFB1,TGFB2,THBD,MAPK14,PRKCA,PRKCE,MAPK1,MAPK3
Regulation of Telomerase	1.98E-5	1.08E-4	8	MYC,CDKN1B,SP1,WT1,TGFB1,EGFR,MAPK1,MAPK3
Small cell lung cancer	2.00E-5	1.08E-4	9	MYC,CDKN1B,ITGA6,ITGB1,PTGS2,PTK2,PIK3CA,LAMA5,BIRC2
ECM-receptor interaction	2.00E-5	1.08E-4	9	ITGA6,ITGA5,ITGB1,ITGB3,ITGB4,SDC1,VTN,LAMA5,CD44
Estrogen signaling pathway	2.06E-5	1.09E-4	5	SP1,BRAF,PIK3CA,MAPK14,MAPK1
p38 signaling mediated by MAPKAP kinases	2.06E-5	1.09E-4	5	SRF,HSPB1,RAF1,YWHAZ,MAPK14
Ras-Independent pathway in NK cell-mediated cytotoxicity	2.06E-5	1.09E-4	5	ITGB1,PIK3CA,RAC1,PAK1,MAPK3
Prolonged ERK activation events	2.06E-5	1.09E-4	5	BRAF,KRAS,RAF1,MAPK1,MAPK3
EPHB forward signaling	2.11E-5	1.12E-4	6	PIK3CA,RAC1,PAK1,ROCK1,MAPK1,MAPK3
Malaria	2.23E-5	1.18E-4	7	HGF,MET,SDC1,TGFB1,TGFB2,TNF,IL1B
Epithelial cell signaling in Helicobacter pylori infection	2.47E-5	1.30E-4	8	MET,PTPRZ1,ADAM17,RAC1,HBEGF,MAPK14,EGFR,PAK1
Gastrin-CREB signalling pathway via PKC and MAPK	2.59E-5	1.36E-4	14	PIK3CA,MMP3,KRAS,RAF1,ANXA1,HBEGF,EDN1,EDNRA,EGFR,PRKCA,PRKCE,MAPK1,MAPK3,MAPK7
IGF-1 Signaling Pathway	2.66E-5	1.38E-4	5	SRF,PIK3CA,RAF1,IGF1R,MAPK3
SHC1 events in ERBB4 signaling	2.66E-5	1.38E-4	5	KRAS,RAF1,HBEGF,MAPK1,MAPK3

TGF-beta receptor signaling	2.91E-5	1.50E-4	7	DAB2,AXIN1,ZFYVE9,YAP1,CAV1,TGFB1,TGFBR3
Genes related to the insulin receptor pathway	2.91E-5	1.50E-4	7	PIK3CA,RAF1,YWHAZ,GSK3A,GSK3B,MAPK1,MAPK3
ErbB2/ErbB3 signaling events	3.00E-5	1.54E-4	6	ERBB2,STAT3,PIK3CA,RAF1,MAPK1,MAPK3
Alzheimer disease-presenilin pathway	3.04E-5	1.55E-4	10	WNT3A,ADAM17,MMP7,MMP13,MMP14,NOTCH2,WNT1,GSK3B,CD44,LRP6
Paxillin-independent events mediated by a4b1 and a4b7	3.39E-5	1.73E-4	5	EPO,ITGB1,PIK3CA,RAC1,CD44
Adrenergic Pathway	3.55E-5	1.80E-4	6	BRAF,PIK3CA,RAF1,MAPK14,EGFR,MAPK1
fMLP induced chemokine gene expression in HMC-1 cells	4.18E-5	2.10E-4	6	RAC1,RAF1,MAPK14,PAK1,MAPK1,MAPK3
Signalling to ERKs	4.18E-5	2.10E-4	6	BRAF,KRAS,RAF1,MAPK14,MAPK1,MAPK3
RANKL/RANK Signaling Pathway	4.26E-5	2.13E-4	7	PTK2,GAB2,FHL2,RAC1,MAPK14,MAPK1,MAPK3
Phosphoinositides and their downstream targets.	4.27E-5	2.13E-4	5	JAG1,RAC1,GSK3A,GSK3B,PRKCE
Multiple antiapoptotic pathways from IGF-1R signaling lead to BAD phosphorylation	4.27E-5	2.13E-4	5	PIK3CA,RAF1,IGF1R,MAPK1,MAPK3
Pertussis	5.08E-5	2.52E-4	8	ITGA5,ITGB1,CXCL5,MAPK14,TNF,MAPK1,MAPK3,IL1B
S1P2 pathway	5.32E-5	2.60E-4	5	RAC1,MAPK14,PAK1,MAPK1,MAPK3
Regulation of eIF4e and p70 S6 Kinase	5.32E-5	2.60E-4	5	PIK3CA,MAPK14,PRKCA,MAPK1,MAPK3
Dorso-ventral axis formation	5.32E-5	2.60E-4	5	NOTCH2,KRAS,EGFR,MAPK1,MAPK3
IL-9 Signaling Pathway	5.32E-5	2.60E-4	5	STAT3,STAT5A,STAT5B,MAPK1,MAPK3
Neurotrophic factor-mediated Trk receptor signaling	5.42E-5	2.64E-4	7	STAT3,GAB2,PIK3CA,RAC1,PAK1,MAPK1,MAPK3
FRS2-mediated cascade	5.69E-5	2.76E-4	6	FGFR1,FGFR2,KRAS,RAF1,MAPK1,MAPK3
Cellular responses to stress	6.32E-5	3.04E-4	13	CDKN1B,EPO,SP1,ERF,EZH2,STAT3,BMI1,VEGFA,MAPK14,MAPK1,MAPK3,MAPK7,EPAS1
HTLV-I infection	6.55E-5	3.12E-4	15	MYC,SRF,MAP3K3,STAT5A,STAT5B,PIK3CA,WNT3A,KRAS,WNT1,GSK3B,FOSL1,TGFB1,TGFB2,EGR1,TNF
Retinoic acid receptors-mediated signaling	6.56E-5	3.12E-4	5	VDR,MAPK14,PRKCA,MAPK1,MAPK3
mCalpain and friends in Cell motility	6.56E-5	3.12E-4	5	ITGB1,PTK2,EGFR,MAPK1,MAPK3

p38 MAPK Signaling Pathway	6.60E-5	3.13E-4	6	MYC,HSPB1,RAC1,TGFB1,TGFB2,MAPK14
C-MYB transcription factor network	6.74E-5	3.19E-4	8	MYC,CDKN1B,SP1,GATA3,PTGS2,KIT,ANPEP,WNT1
B cell activation	6.81E-5	3.21E-4	7	MAP3K3,PIK3CA,RAC1,RAF1,MAPK14,MAPK1,MAPK3
Disease	6.83E-5	3.21E-4	37	MYC,CDKN1B,SP1,ERBB2,PAG1,STAT3,GAB2,STAT5A,STAT5B,PARP1,ZFYVE9,BRAF,PIK3CA,CXCR4,JAG1,FGFR1,ADAM17,FGFR2,KIT,SDC1,RAC1,KRAS,RAF1,HBEGF,WWTR1,GSK3A,GSK3B,GSN,TGFB1,EGFR,CD44,PRKCA,PRKCE,MAPK1,MAPK3,HAS2,CUX1
Viral carcinogenesis	7.34E-5	3.44E-4	13	CDKN1B,SRF,STAT3,STAT5A,STAT5B,PIK3CA,RAC1,KRAS,YWHAZ,GSN,SCRIB,MAPK1,MAPK3
Regulation of BAD phosphorylation	8.00E-5	3.73E-4	5	PIK3CA,KIT,IGF1R,MAPK1,MAPK3
mTOR signaling pathway	8.50E-5	3.90E-4	7	BRAF,PIK3CA,VEGFA,PRKCA,TNF,MAPK1,MAPK3
Long-term depression	8.50E-5	3.90E-4	7	BRAF,KRAS,RAF1,IGF1R,PRKCA,MAPK1,MAPK3
Integrated Breast Cancer Pathway	8.86E-5	4.05E-4	11	MYC,SP1,BRAF,RAC1,VEGFA,KRAS,ANXA1,GSK3A,EGFR,PAK1,MAPK1
T cell receptor signaling pathway	9.08E-5	4.14E-4	9	PIK3CA,KRAS,RAF1,GSK3B,MAPK14,PAK1,TNF,MAPK1,MAPK3
mTOR signaling pathway	9.46E-5	4.30E-4	7	BRAF,RAC1,RAF1,YWHAZ,PRKCA,MAPK1,MAPK3
Glucocorticoid receptor regulatory network	9.65E-5	4.37E-4	8	GATA3,STAT5A,STAT5B,GSK3B,MAPK14,EGR1,MAPK1,MAPK3
Signalling to RAS	9.68E-5	4.37E-4	5	KRAS,RAF1,MAPK14,MAPK1,MAPK3
Hypertrophic cardiomyopathy (HCM)	1.05E-4	4.73E-4	8	ITGA6,ITGA5,ITGB1,ITGB3,ITGB4,TGFB1,TGFB2,TNF
FSH signaling pathway	1.16E-4	5.16E-4	5	RAF1,MAPK14,PRKCA,MAPK1,MAPK3
B Cell Receptor Signaling Pathway	1.18E-4	5.22E-4	11	PTK2,STAT3,GAB2,BRAF,RAF1,GSK3A,GSK3B,MAPK14,PRKCE,MAPK1,MAPK3
Delta-Notch Signaling Pathway	1.25E-4	5.49E-4	8	STAT3,JAG1,ADAM17,NOTCH2,GSK3B,EGFR,MAPK1,MAPK3
IL-4 signaling Pathway	1.29E-4	5.66E-4	7	PTK2,STAT5A,PIK3CA,CXCR4,MAPK14,MAPK1,MAPK3
Thromboxane A2 receptor signaling	1.30E-4	5.70E-4	6	RAC1,MAPK14,EGFR,PRKCA,PRKCE,ROCK1
Amoebiasis	1.31E-4	5.71E-4	9	PTK2,PIK3CA,HSPB1,LAMA5,TGFB1,TGFB2,PRKCA,TNF,IL1B
Progesterone-mediated oocyte maturation	1.35E-4	5.87E-4	8	BRAF,PIK3CA,KRAS,RAF1,MAPK14,IGF1R,MAPK1,MAPK3
IRS-related events triggered by IGF1R	1.35E-4	5.87E-4	8	PIK3CA,FGFR1,FGFR2,KRAS,RAF1,IGF1R,MAPK1,MAPK3
Natural killer cell mediated cytotoxicity	1.38E-4	5.95E-4	10	BRAF,PIK3CA,RAC1,KRAS,RAF1,PAK1,PRKCA,TNF,MAPK1,MAPK3

Sema4D in semaphorin signaling	1.38E-4	5.95E-4	5	ERBB2,MET,RAC1,ROCK2,ROCK1
VEGF, Hypoxia, and Angiogenesis	1.38E-4	5.95E-4	5	PTK2,PIK3CA,VEGFA,FLT1,PRKCA
Alzheimer disease-amyloid secretase pathway	1.42E-4	6.09E-4	7	ADAM17,MAPK14,PRKCA,PRKCE,MAPK1,MAPK3,MAPK7
Inflammation mediated by chemokine and cytokine signaling pathway	1.46E-4	6.22E-4	12	ITGB1,STAT3,PIK3CA,CXCR4,RAC1,KRAS,RAF1,PAK1,PRKCE,ROCK1,MAPK1,MAPK3
Collagen formation	1.47E-4	6.22E-4	8	ITGA6,ITGB4,LOXL3,MMP3,MMP7,MMP13,COL8A1,COL8A2
Members of the BCR signaling pathway	1.47E-4	6.22E-4	6	PIK3CA,RAF1,GSK3A,GSK3B,MAPK1,MAPK3
canonical Wnt signaling	1.47E-4	6.22E-4	6	AXIN1,AXIN2,WNT3A,WNT1,GSK3B,LRP6
BCR signaling pathway	1.57E-4	6.59E-4	7	PAG1,PIK3CA,RAC1,RAF1,MAPK14,MAPK1,MAPK3
Genes related to Wnt-mediated signal transduction	1.72E-4	7.16E-4	8	MYC,AXIN1,WNT3A,WNT1,GSK3A,GSK3B,FOSL1,LRP6
Signaling by Type 1 Insulin-like Growth Factor 1 Receptor (IGF1R)	1.72E-4	7.16E-4	8	PIK3CA,FGFR1,FGFR2,KRAS,RAF1,IGF1R,MAPK1,MAPK3
IGF1R signaling cascade	1.72E-4	7.16E-4	8	PIK3CA,FGFR1,FGFR2,KRAS,RAF1,IGF1R,MAPK1,MAPK3
Kit Receptor Signaling Pathway	1.72E-4	7.16E-4	7	STAT3,STAT5A,STAT5B,KIT,RAF1,PRKCA,MAPK1
Dilated cardiomyopathy	1.86E-4	7.71E-4	8	ITGA6,ITGA5,ITGB1,ITGB3,ITGB4,TGFB1,TGFB2,TNF
Calcineurin-regulated NFAT-dependent transcription in lymphocytes	1.88E-4	7.75E-4	6	GATA3,PTGS2,PPARG,FOSL1,EGR1,TNF
Matrix Metalloproteinases	1.92E-4	7.85E-4	5	MMP3,MMP7,MMP13,MMP14,TNF
IL17 signaling pathway	1.92E-4	7.85E-4	5	SP1,STAT3,GSK3B,MAPK1,MAPK3
Downstream Signaling Events Of B Cell Receptor (BCR)	2.24E-4	9.09E-4	11	CDKN1B,ERBB2,PIK3CA,FGFR1,FGFR2,KIT,KRAS,HBEGF,GSK3A,GSK3B,EGFR
Monoamine Transport	2.25E-4	9.09E-4	5	ITGB3,TGFB1I1,MAPK14,TNF,IL1B
Trk receptor signaling mediated by PI3K and PLC-gamma	2.25E-4	9.09E-4	5	STAT5A,PIK3CA,YWHAZ,GSK3B,EGR1
Rheumatoid arthritis	2.34E-4	9.42E-4	8	MMP3,CXCL5,VEGFA,FLT1,TGFB1,TGFB2,TNF,IL1B
Endocytosis	2.57E-4	1.02E-3	12	DAB2,MET,ZFYVE9,CXCR4,FGFR2,KIT,FLT1,CAV1,TGFB1,TGFB2,IGF1R,EGFR

Syndecan-2-mediated signaling events	2.61E-4	1.03E-3	5	ITGA5,ITGB1,TGFB1,MAPK1,MAPK3
Signal transduction through IL1R	2.61E-4	1.03E-3	5	TGFB1,TGFB2,MAPK14,TNF,IL1B
Role of Calcineurin-dependent NFAT signaling in lymphocytes	2.63E-4	1.03E-3	6	YWHAZ,GSK3B,MAPK14,PRKCA,PRKCE,MAPK3
Hedgehog signaling pathway	2.63E-4	1.03E-3	6	BMP2,BMP4,WNT3A,WNT1,SHH,GSK3B
B cell receptor signaling pathway	2.71E-4	1.06E-3	7	PIK3CA,RAC1,KRAS,RAF1,GSK3B,MAPK1,MAPK3
Vascular smooth muscle contraction	2.89E-4	1.12E-3	9	BRAF,ROCK2,RAF1,EDNRA,PRKCA,PRKCE,ROCK1, MAPK1,MAPK3
p38 MAPK Signaling Pathway	3.02E-4	1.15E-3	5	MYC,HSPB1,RAC1,TGFB2,MAPK14
Validated transcriptional targets of AP1 family members Fra1 and Fra2	3.02E-4	1.15E-3	5	SP1,ITGB4,PLAUR,FOSL1,THBD
CXCR3-mediated signaling events	3.02E-4	1.15E-3	5	PIK3CA,RAF1,MAPK14,MAPK1,MAPK3
Wnt/beta-catenin Pathway	3.02E-4	1.15E-3	5	AXIN1,AXIN2,PIN1,GSK3A,GSK3B
Class I PI3K signaling events mediated by Akt	3.02E-4	1.15E-3	5	CDKN1B,RAF1,YWHAZ,GSK3A,GSK3B
Apoptotic execution phase	3.26E-4	1.24E-3	6	HMGB1,PTK2,VIM,BIRC2,GSN,ROCK1
FCERI mediated MAPK activation	3.26E-4	1.24E-3	6	RAC1,KRAS,RAF1,PAK1,MAPK1,MAPK3
Cell surface interactions at the vascular wall	3.35E-4	1.27E-3	8	ITGA6,ITGA5,ITGB1,ITGB3,PIK3CA,KRAS,CAV1,TH BD
BCR Signaling Pathway	3.47E-4	1.30E-3	5	RAC1,RAF1,MAPK14,PRKCA,MAPK3
Genes related to regulation of the actin cytoskeleton	3.47E-4	1.30E-3	5	FSCN2,ROCK2,PAK1,ROCK1,FSCN1
G alpha i Pathway	3.47E-4	1.30E-3	5	STAT3,BRAF,RAF1,EGFR,MAPK1
Endothelin	3.47E-4	1.30E-3	5	RAF1,EDN1,EDNRA,PRKCA,MAPK1
Angiotensin II-stimulated signaling through G proteins and beta-arrestin	3.47E-4	1.30E-3	5	RAF1,EGR1,PRKCA,MAPK1,MAPK3
PDGFR-beta signaling pathway	3.62E-4	1.35E-3	6	MYC,STAT3,STAT5A,STAT5B,PIK3CA,RAC1
Bacterial invasion of epithelial cells	3.79E-4	1.40E-3	7	ITGA5,ITGB1,PTK2,MET,PIK3CA,RAC1,CAV1
T cell activation	3.79E-4	1.40E-3	7	BRAF,PIK3CA,RAC1,RAF1,PAK1,MAPK1,MAPK3

Estrogen signaling pathway	3.85E-4	1.42E-3	8	SP1,PIK3CA,KRAS,RAF1,HBEGF,EGFR,MAPK1,MAPK3
Pathogenic Escherichia coli infection	4.00E-4	1.47E-3	6	ITGB1,ROCK2,YWHAZ,CLDN1,PRKCA,ROCK1
ECM proteoglycans	4.00E-4	1.47E-3	6	ITGB1,ITGB3,VTN,LAMA5,TGFB1,TGFB2
Regulation of retinoblastoma protein	4.41E-4	1.60E-3	6	CDKN1B,MET,RAF1,PPARG,TGFB2,MAPK14
Pathogenic Escherichia coli infection	4.41E-4	1.60E-3	6	ITGB1,ROCK2,YWHAZ,CLDN1,PRKCA,ROCK1
IL23-mediated signaling events	4.53E-4	1.64E-3	5	STAT3,STAT5A,PIK3CA,TNF,IL1B
NOD-like receptor signaling pathway	4.86E-4	1.75E-3	6	BIRC2,MAPK14,TNF,MAPK1,MAPK3,IL1B
Coregulation of Androgen receptor activity	4.86E-4	1.75E-3	6	KDM3A,SRF,FHL2,GSN,TGFB1I1,PRDX1
Chagas disease (American trypanosomiasis)	5.01E-4	1.80E-3	8	PIK3CA,TGFB1,TGFB2,MAPK14,TNF,MAPK1,MAPK3,IL1B
Signaling events regulated by Ret tyrosine kinase	5.14E-4	1.83E-3	5	PIK3CA,RAC1,PRKCA,MAPK1,MAPK3
Collagen degradation	5.14E-4	1.83E-3	5	ADAM17,MMP3,MMP7,MMP13,MMP14
SIDS Susceptibility Pathways	5.44E-4	1.90E-3	10	SP1,GATA3,VEGFA,FOXM1,YBX1,POU5F1,YWHAZ,EGR1,TNF,IL1B
FOXM1 transcription factor network	5.81E-4	2.02E-3	5	MYC,SP1,FOXM1,GSK3A,TGFA
Apoptotic cleavage of cellular proteins	5.81E-4	2.02E-3	5	PTK2,VIM,BIRC2,GSN,ROCK1
Aldosterone-regulated sodium reabsorption	5.81E-4	2.02E-3	5	PIK3CA,KRAS,PRKCA,MAPK1,MAPK3
IRS-mediated signalling	6.03E-4	2.08E-3	7	PIK3CA,FGFR1,FGFR2,KRAS,RAF1,MAPK1,MAPK3
IFN-gamma pathway	6.55E-4	2.22E-3	5	STAT3,PIK3CA,MAPK1,MAPK3,IL1B
Nuclear signaling by ERBB4	6.55E-4	2.22E-3	5	STAT5A,STAT5B,YAP1,ADAM17,HBEGF
Interleukin-11 Signaling Pathway	6.55E-4	2.22E-3	5	STAT3,RAF1,TGFB1,MAPK1,MAPK3
Cell junction organization	6.98E-4	2.36E-3	7	ITGA6,ITGB1,ITGB4,CLDN4,CLDN1,LIMS1,CTNND1
RhoA signaling pathway	7.35E-4	2.48E-3	5	CDKN1B,SRF,ROCK2,CYR61,ROCK1
IRS-related events	7.49E-4	2.52E-3	7	PIK3CA,FGFR1,FGFR2,KRAS,RAF1,MAPK1,MAPK3
Salmonella infection	8.04E-4	2.68E-3	7	RAC1,ROCK2,MAPK14,ROCK1,MAPK1,MAPK3,IL1B
miRs in Muscle Cell Differentiation	8.23E-4	2.72E-3	5	SRF,EZH2,ID2,PRKCA,PRKCE
Negative regulation of FGFR signaling	8.23E-4	2.72E-3	5	BRAF,FGFR1,FGFR2,MAPK1,MAPK3

IL4-mediated signaling events	8.34E-4	2.75E-3	6	SP1,ITGB3,STAT5A,STAT5B,PIK3CA,MAPK14
NCAM signaling for neurite out-growth	8.34E-4	2.75E-3	6	PTK2,FGFR1,KRAS,RAF1,MAPK1,MAPK3
FOXA1 transcription factor network	9.18E-4	3.00E-3	5	CDKN1B,SP1,FOXA1,VTN,SHH
IL6-mediated signaling events	9.18E-4	3.00E-3	5	MYC,STAT3,PIK3CA,RAC1,MAPK14
Epstein-Barr virus infection	9.57E-4	3.11E-3	11	MYC,CDKN1B,STAT3,PIK3CA,HSPB1,VIM,YWHAZ,GSK3B,MAPK14,CSNK2B,CD44
LPA receptor mediated events	9.85E-4	3.18E-3	6	PTK2,RAC1,HBEGF,GSK3B,EGFR,PRKCE
Fas Signaling Pathway	9.85E-4	3.18E-3	6	MET,PARP1,HSPB1,EGFR,ROCK1,MAPK1
Gap junction	9.86E-4	3.18E-3	7	KRAS,RAF1,EGFR,PRKCA,MAPK1,MAPK3,MAPK7
Degradation of the extracellular matrix	9.86E-4	3.18E-3	7	ADAM17,MMP3,MMP7,MMP13,MMP14,LAMA5,CD44
Ceramide signaling pathway	1.02E-3	3.27E-3	5	MYC,RAF1,TNF,MAPK1,MAPK3
TNF receptor signaling pathway	1.02E-3	3.27E-3	5	MAP3K3,ADAM17,BIRC2,CAV1,TNF
Wnt signaling pathway	1.04E-3	3.31E-3	14	MYC,MYCN,AXIN1,AXIN2,WNT3A,MMP7,WNT1,EDN1,GSK3B,CSNK2B,LRP6,PRKCA,PRKCE,TNF
Insulin receptor signalling cascade	1.05E-3	3.32E-3	7	PIK3CA,FGFR1,FGFR2,KRAS,RAF1,MAPK1,MAPK3
Integrin cell surface interactions	1.07E-3	3.34E-3	6	ITGA6,ITGA5,ITGB1,ITGB3,VTN,CD44
p53 pathway feedback loops 2	1.13E-3	3.52E-3	5	MYC,PIK3CA,KRAS,MAPK14,TP63
Differentiation Pathway in PC12 Cells; this is a specific case of PAC1 Receptor Pathway.	1.13E-3	3.52E-3	5	BRAF,PIK3CA,EGR1,MAPK1,MAPK3
Long-term potentiation	1.16E-3	3.59E-3	6	BRAF,KRAS,RAF1,PRKCA,MAPK1,MAPK3
Inflammatory bowel disease (IBD)	1.35E-3	4.15E-3	6	GATA3,STAT3,TGFB1,TGFB2,TNF,IL1B
T Cell Receptor Signaling Pathway	1.38E-3	4.21E-3	5	PIK3CA,RAC1,RAF1,PRKCA,MAPK3
Cellular Senescence	1.46E-3	4.42E-3	10	CDKN1B,SP1,ERF,EZH2,STAT3,BMI1,MAPK14,MAPK1,MAPK3,MAPK7
Type II diabetes mellitus	1.52E-3	4.59E-3	5	PIK3CA,PRKCE,TNF,MAPK1,MAPK3
MyD88-independent cascade	1.63E-3	4.87E-3	7	HMGB1,AGER,BIRC2,MAPK14,MAPK1,MAPK3,MAPK7
Toll Like Receptor 3 (TLR3) Cascade	1.63E-3	4.87E-3	7	HMGB1,AGER,BIRC2,MAPK14,MAPK1,MAPK3,MAPK7
Signaling by the B Cell Receptor (BCR)	1.64E-3	4.87E-3	11	CDKN1B,ERBB2,PIK3CA,FGFR1,FGFR2,KIT,KRAS,HBEGF,GSK3A,GSK3B,EGFR
Interferon type I	1.67E-3	4.87E-3	5	STAT3,GAB2,STAT5A,RAC1,MAPK14

Interleukin-3, 5 and GM-CSF signaling	1.67E-3	4.87E-3	5	GAB2,STAT5A,STAT5B,PIK3CA,YWHAZ
Signaling by TGF-beta Receptor Complex	1.68E-3	4.87E-3	6	MYC,SP1,PARP1,ZFYVE9,WWTR1,TGFB1
Loss of Function of SMAD4 in Cancer	1.68E-3	4.87E-3	6	MYC,SP1,PARP1,ZFYVE9,WWTR1,TGFB1
TGFBR2 MSI Frameshift Mutants in Cancer	1.68E-3	4.87E-3	6	MYC,SP1,PARP1,ZFYVE9,WWTR1,TGFB1
Loss of Function of TGFBR2 in Cancer	1.68E-3	4.87E-3	6	MYC,SP1,PARP1,ZFYVE9,WWTR1,TGFB1
Loss of Function of SMAD2/3 in Cancer	1.68E-3	4.87E-3	6	MYC,SP1,PARP1,ZFYVE9,WWTR1,TGFB1
TGFBR2 Kinase Domain Mutants in Cancer	1.68E-3	4.87E-3	6	MYC,SP1,PARP1,ZFYVE9,WWTR1,TGFB1
Loss of Function of TGFBR1 in Cancer	1.68E-3	4.87E-3	6	MYC,SP1,PARP1,ZFYVE9,WWTR1,TGFB1
TGFBR1 LBD Mutants in Cancer	1.68E-3	4.87E-3	6	MYC,SP1,PARP1,ZFYVE9,WWTR1,TGFB1
SMAD2/3 Phosphorylation Motif Mutants in Cancer	1.68E-3	4.87E-3	6	MYC,SP1,PARP1,ZFYVE9,WWTR1,TGFB1
TGFBR1 KD Mutants in Cancer	1.68E-3	4.87E-3	6	MYC,SP1,PARP1,ZFYVE9,WWTR1,TGFB1
Signaling by TGF-beta Receptor Complex in Cancer	1.68E-3	4.87E-3	6	MYC,SP1,PARP1,ZFYVE9,WWTR1,TGFB1
SMAD2/3 MH2 Domain Mutants in Cancer	1.68E-3	4.87E-3	6	MYC,SP1,PARP1,ZFYVE9,WWTR1,TGFB1
SMAD4 MH2 Domain Mutants in Cancer	1.68E-3	4.87E-3	6	MYC,SP1,PARP1,ZFYVE9,WWTR1,TGFB1
Caspase cascade in apoptosis	1.83E-3	5.23E-3	5	PARP1,VIM,BIRC2,GSN,TNF
Regulation of toll-like receptor signaling pathway	2.18E-3	6.11E-3	7	PIK3CA,RAC1,MAPK14,TNF,MAPK1,MAPK3,IL1B
Apoptosis signaling pathway	2.18E-3	6.11E-3	7	PIK3CA,BIRC2,PRKCA,PRKCE,TNF,MAPK1,MAPK3
Id Signaling Pathway	2.18E-3	6.11E-3	5	BMP2,VEGFA,FLT1,ID2,IGF1R
Toll-like receptor signaling pathway	2.31E-3	6.45E-3	7	PIK3CA,RAC1,MAPK14,TNF,MAPK1,MAPK3,IL1B
Apoptosis	2.33E-3	6.46E-3	9	HMGB1,PTK2,ADAM17,VIM,BIRC2,YWHAZ,GSN,ROCK1,TNF
Fcgamma receptor (FCGR) dependent phagocytosis	2.57E-3	7.10E-3	7	PTK2,PIK3CA,RAC1,PAK1,PRKCE,MAPK1,MAPK3

Measles	2.59E-3	7.12E-3	8	CDKN1B,STAT3,STAT5A,STAT5B,PIK3CA,GSK3B,CSNK2B,IL1B
Alzheimers Disease	2.70E-3	7.37E-3	6	ADAM17,GSK3B,TNF,MAPK1,MAPK3,IL1B
Regulation of Wnt-mediated beta catenin signaling and target gene transcription	2.70E-3	7.37E-3	6	MYC,AXIN2,ID2,YWHAZ,CYR61,SNAI2
Toll-like receptor signaling pathway	2.71E-3	7.38E-3	7	PIK3CA,RAC1,MAPK14,TNF,MAPK1,MAPK3,IL1B
ATF-2 transcription factor network	2.80E-3	7.58E-3	5	TGFB2,MAPK14,PRKCA,MAPK1,MAPK3
RAC1 signaling pathway	2.80E-3	7.58E-3	5	STAT3,STAT5A,RAC1,MAPK14,PAK1
TRAF6 mediated induction of NFkB and MAP kinases upon TLR7/8 or 9 activation	3.46E-3	9.13E-3	6	HMGB1,AGER,MAPK14,MAPK1,MAPK3,MAPK7
HIV-I Nef: negative effector of Fas and TNF	3.53E-3	9.29E-3	5	PTK2,PARP1,BIRC2,GSN,TNF
Fc-epsilon receptor I signaling in mast cells	3.80E-3	9.87E-3	5	GAB2,PIK3CA,RAF1,MAPK1,MAPK3

Supplementary Table S3: The 71 EMT-implicated genes with concordance between CNG and increased gene expression

GeneID	Symbol	Number of samples with CNG	Alias	Cytoband	FullName	GeneType
7534	YWHAZ	345	14-3-3-zeta|KCIP-1|YWHAD	8q23.1	tyrosine 3-monooxygenase/ tryptophan 5-monooxygenase activation protein, zeta polypeptide	protein-coding
5290	PIK3CA	313	PI3K|p110-alpha	3q26.3	phosphoinositide-3-kinase, catalytic, alpha polypeptide	protein-coding
92140	MTDH	293	3D3|AEG-1|AEG1|LYRIC|LYRIC/3D3	8q22.1	metadherin	protein-coding
1956	EGFR	285	ERBB|ERBB1|HER1 |PIG61|mENA	7p12	epidermal growth factor receptor	protein-coding
1894	ECT2	271	ARHGEF31	3q26.1-q26.2	epithelial cell transforming sequence 2 oncogene	protein-coding
2064	ERBB2	220	CD340|HER-2|HER-2/ neu|HER2|MLN 19|NEU| NGL|TKR1	17q11.2-q12|17q21.1	v-erb-b2 erythroblastic leukemia viral oncogene homolog 2, neuro/glioblastoma derived oncogene homolog (avian)	protein-coding
54845	ESRP1	219	RBM35A|RMB35A	8q22.1	epithelial splicing regulatory protein 1	protein-coding
23513	SCRIB	157	CRIB1|SCRB1|SCRIB1|Vartul	8q24.3	scribbled homolog (Drosophila)	protein-coding
10397	NDRG1	151	CAP43|CMT4D|DRG1|GC4| HMSNL|NDR1|NMSL|PROXY1 |RIT42|RTP|TARG1|TDD5	8q24.3	N-myc downstream regulated 1	protein-coding
56648	EIF5A2	150	EIF-5A2|eIF5AII	3q26.2	eukaryotic translation initiation factor 5A2	protein-coding
7764	ZNF217	145	ZABC1	20q13.2	zinc finger protein 217	protein-coding
3845	KRAS	138	C-K-RAS|K-RAS2A|K-RAS2B|K-RAS4A|K-RAS4B| KI-RAS|KRAS1|KRAS2|NS |NS3|RASK2	12p12.1	v-Ki-ras2 Kirsten rat sarcoma viral oncogene homolog	protein-coding
4609	MYC	135	MRTL|bHLHe39|c-Myc	8q24.21	v-myc myelocytomatosis viral oncogene homolog (avian)	protein-coding
8626	TP63	113	AIS|B(p51A)|B(p51B)|EEC3|KET|LMS|NBP|OFC8|RHS|SHFM4| TP53CP|TP53L|TP73L|p40|p51|p53CP|p63|p73H|p73L	3q28	tumor protein p63	protein-coding
5058	PAK1	99	PAKalpha	11q13-q14	p21 protein (Cdc42/ Rac)-activated kinase 1	protein-coding

142	PARP1	94	ADPRT|ADPRT 1|ADPRT1|PARP|PARP-1|PPOL|pADPRT-1	1q41-q42	poly (ADP-ribose) polymerase 1	protein-coding
2260	FGFR1	93	BFGFR|CD331|CEK|FGFBR |FGFR-1|FLG|FLT-2|FLT2|HBGFR|KAL2|N-SAM|OGD|bFGF-R-1	8p12	fibroblast growth factor receptor 1	protein-coding
7227	TRPS1	89	GC79|LGCR	8q24.12	trichorhinophalangeal syndrome I	protein-coding
9846	GAB2	86	-	11q14.1	GRB2-associated binding protein 2	protein-coding
10413	YAP1	83	YAP|YAP2|YAP65|YKI	11q13	Yes-associated protein 1	protein-coding
329	BIRC2	80	API1|HIAP2|Hiap-2|MIHB |RNF48|c-IAP1|cIAP1	11q22	baculoviral IAP repeat containing 2	protein-coding
25937	WWTR1	79	TAZ	3q23-q24	WW domain containing transcription regulator 1	protein-coding
9076	CLDN1	79	CLD1|ILVASC|SEMP1	3q28-q29	claudin 1	protein-coding
2305	FOXM1	71	FKHL16|FOXM1B|HFH-11|HFH11|HNF-3|INS-1|MPHOSPH2 |MPP-2|MPP2|PIG29|TGT3|TRIDENT	12p13	forkhead box M1	protein-coding
4040	LRP6	62	ADCAD2	12p13.2	low density lipoprotein receptor-related protein 6	protein-coding
5879	RAC1	59	Rac-1|TC-25|p21-Rac1	7p22	ras-related C3 botulinum toxin substrate 1 (rho family, small GTP binding protein Rac1)	protein-coding
55824	PAG1	58	CBP|PAG	8q21.13	phosphoprotein associated with glycosphingolipid microdomains 1	protein-coding
6722	SRF	50	MCM1	6p21.1	serum response factor (c-fos serum response element-binding transcription factor)	protein-coding
1655	DDX5	50	G17P1|HLR1|HUMP68|p68	17q21	DEAD (Asp-Glu-Ala-Asp) box polypeptide 5	protein-coding
3146	HMGB1	49	HMG1|HMG3|SBP-1	13q12	high mobility group box 1	protein-coding
4215	MAP3K3	47	MAPKKK3|MEKK3	17q23.3	mitogen-activated protein kinase kinase kinase 3	protein-coding
4233	MET	45	AUTS9|HGFR|RCCP2|c-Met	7q31	met proto-oncogene (hepatocyte growth factor receptor)	protein-coding
1522	CTSZ	43	CTSX	20q13.32	cathepsin Z	protein-coding

3480	IGF1R	40	CD221|IGFIR|IGFR|JTK13	15q26.3	insulin-like growth factor 1 receptor	protein-coding
3911	LAMA5	38	-	20q13.2-q13.3	laminin, alpha 5	protein-coding
5300	PIN1	37	DOD|UBL5	19p13	peptidylprolyl cis/ trans isomerase, NIMA-interacting 1	protein-coding
10049	DNAJB6	37	DJ4|DnaJ|HHDJ1|HSJ-2|HSJ2|MRJ|MSJ-1	7q36.3	DnaJ (Hsp40) homolog, subfamily B, member 6	protein-coding
1523	CUX1	37	CASP|CDP|CDP/Cut|CDP1|COY1|CUTL1|CUX|Clox|Cux/CDP|GOLIM6|Nbla10317|p100|p110|p200|p75	7q22.1	cut-like homeobox 1	protein-coding
7422	VEGFA	36	MVCD1|VEGF|VPF	6p12	vascular endothelial growth factor A	protein-coding
960	CD44	36	CDW44|CSPG8|ECMR-III|HCELL|HUTCH-I|IN|LHR|MC56|MDU2|MDU3|MIC4|Pgp1	11p13	CD44 molecule (Indian blood group)	protein-coding
3169	FOXA1	35	HNF3A|TCF3A	14q12-q13	forkhead box A1	protein-coding
1027	CDKN1B	35	CDKN4|KIP1|MEN1B|MEN4|P27KIP1	12p13.1-p12	cyclin-dependent kinase inhibitor 1B (p27, Kip1)	protein-coding
1748	DLX4	34	BP1|DLX7|DLX8|DLX9	17q21.33	distal-less homeobox 4	protein-coding
29126	CD274	34	B7-H|B7H1|PD-L1|PDCD1L1|PDCD1LG1|PDL1	9p24	CD274 molecule	protein-coding
5598	MAPK7	32	BMK1|ERK4|ERK5|PRKM7	17p11.2	mitogen-activated protein kinase 7	protein-coding
673	BRAF	32	B-RAF1|BRAF1|NS7|RAFB1	7q34	v-raf murine sarcoma viral oncogene homolog B1	protein-coding
6591	SNAI2	31	SLUG|SLUGH1|SNAIL2|WS2D	8q11	snail homolog 2 (Drosophila)	protein-coding
3037	HAS2	31	-	8q24.12	hyaluronan synthase 2	protein-coding
6624	FSCN1	31	FAN1|HSN|SNL|p55	7p22	fascin homolog 1, actin-bundling protein (Strongylocentrotus purpuratus)	protein-coding
4904	YBX1	29	BP-8|CSDA2|CSDB|DBPB|MDR-NF1|NSEP-1|NSEP1|YB-1|YB1	1p34	Y box binding protein 1	protein-coding
5894	RAF1	27	CRAF|NS5|Raf-1|c-Raf	3p25	v-raf-1 murine leukemia viral oncogene homolog 1	protein-coding
2931	GSK3A	27	-	19q13.2	glycogen synthase kinase 3 alpha	protein-coding
57118	CAMK1D	27	CKLiK|CaM-K1|CaMKID	10p13	calcium/calmodulin-dependent protein kinase ID	protein-coding
51053	GMNN	26	Gem	6p22.3	geminin, DNA replication inhibitor	protein-coding

2146	EZH2	25	ENX-1|ENX1|EZH1|KMT6|KMT6A	7q35-q36	enhancer of zeste homolog 2 (Drosophila)	protein-coding
57496	MKL2	24	MRTF-B|NPD001	16p13.12	MKL/myocardin-like 2	protein-coding
5595	MAPK3	24	ERK-1|ERK1|ERT2|HS44KDAP|HUMKER1A|P44ERK1|P44MAPK|PRKM3|p44-ERK1|p44-MAPK	16p11.2	mitogen-activated protein kinase 3	protein-coding
3756	KCNH1	24	EAG|EAG1|Kv10.1|h-eag	1q32.2	potassium voltage-gated channel, subfamily H (eag-related), member 1	protein-coding
3206	HOXA10	24	HOX1|HOX1.8|HOX1H|PL	7p15.2	homeobox A10	protein-coding
1432	MAPK14	23	CSBP|CSBP1|CSBP2|CSPB1|EXIP|Mxi2|PRKM14|PRKM15|RK|SAPK2A|p38|p38ALPHA	6p21.3-p21.2	mitogen-activated protein kinase 14	protein-coding
2263	FGFR2	23	BEK|BFR-1|CD332|CEK3|CFD1|ECT1|JWS|K-SAM|KGFR|TK14|TK25	10q26	fibroblast growth factor receptor 2	protein-coding
2056	EPO	23	EP|MVCD2	7q22	erythropoietin	protein-coding
1045	CDX2	23	CDX-3|CDX3	13q12.3	caudal type homeobox 2	protein-coding
857	CAV1	23	BSCL3|CGL3|MSTP085|VIP21	7q31.1	caveolin 1, caveolae protein, 22kDa	protein-coding
5594	MAPK1	22	ERK|ERK2|ERT1|MAPK2|P42MAPK|PRKM1|PRKM2|p38|p40|p41|p41mapk	22q11.2|22q11.21	mitogen-activated protein kinase 1	protein-coding
3418	IDH2	22	D2HGA2|ICD-M|IDH|IDHM|IDP|IDPM|mNADP-IDH	15q26.1	isocitrate dehydrogenase 2 (NADP+), mitochondrial	protein-coding
8312	AXIN1	22	AXIN|PPP1R49	16p13.3	axin 1	protein-coding
5744	PTHLH	21	BDE2|HHM|PLP|PTHR|PTHRP	12p12.1-p11.2	parathyroid hormone-like hormone	protein-coding
4585	MUC4	20	ASGP|HSA276359|MUC-4	3q29	mucin 4, cell surface associated	protein-coding
1601	DAB2	20	DOC-2|DOC2	5p13	disabled homolog 2, mitogen-responsive phosphoprotein (Drosophila)	protein-coding
1460	CSNK2B	20	CK2B|CK2N|CSK2B|G5A	6p21-p12|6p21.3	casein kinase 2, beta polypeptide	protein-coding

Supplementary Table S4: Functional enrichment results of the 71 EMT-implicated genes with concordance between CNG and increased gene expression

Supplementary Table S5: The pan-cancer CNV frequency of the 71 EMT-implicated genes with concordance between CNG and increased gene expression

STUDY_ABBREVIATION	STUDY_NAME	NUM_OF_CASES_ALTERED	PERCENT_CASES_ALTERED
Ovarian (TCGA)	Ovarian Serous Cystadenocarcinoma (TCGA, Provisional)	493	85.10%
CCLE (Novartis/Broad 2012)	Cancer Cell Line Encyclopedia (Novartis/ Broad, Nature 2012)	813	81.70%
Ovarian (TCGA pub)	Ovarian Serous Cystadenocarcinoma (TCGA, Nature 2011)	395	80.80%
Esophagus (TCGA)	Esophageal Carcinoma (TCGA, Provisional)	74	80.40%
Lung squ (TCGA)	Lung Squamous Cell Carcinoma (TCGA, Provisional)	398	79.40%
Uterine CS (TCGA)	Uterine Carcinosarcoma (TCGA, Provisional)	44	78.60%
Lung squ (TCGA pub)	Lung Squamous Cell Carcinoma (TCGA, Nature 2012)	131	73.60%
NCI-60	NCI-60 Cell Lines (NCI, Cancer Res. 2012)	42	70%
Head & neck (TCGA pub)	Head and Neck Squamous Cell Carcinoma (TCGA, in revision)	186	66.70%
Sarcoma (TCGA)	Sarcoma (TCGA, Provisional)	170	66.40%
Bladder (TCGA)	Bladder Urothelial Carcinoma (TCGA, Provisional)	270	66.20%
Breast (TCGA)	Breast Invasive Carcinoma (TCGA, Provisional)	710	65.70%
Bladder (TCGA pub)	Bladder Urothelial Carcinoma (TCGA, Nature 2014)	83	64.80%
Lung adeno (TCGA pub)	Lung Adenocarcinoma (TCGA, Nature 2014)	149	64.80%
Stomach (TCGA)	Stomach Adenocarcinoma (TCGA, Provisional)	285	64.60%
Lung adeno (TCGA)	Lung Adenocarcinoma (TCGA, Provisional)	332	64.30%
Head & neck (TCGA)	Head and Neck Squamous Cell Carcinoma (TCGA, Provisional)	335	64.20%
Stomach (TCGA pub)	Stomach Adenocarcinoma (TCGA, Nature 2014)	185	63.10%
Prostate (MICH)	Prostate Adenocarcinoma, Metastatic (Michigan, Nature 2012)	38	62.30%
Breast (TCGA pub)	Breast Invasive Carcinoma (TCGA, Nature 2012)	467	60%
MPNST (MSKCC)	Malignant Peripheral Nerve Sheath Tumor (MSKCC, Nature Genetics 2014)	9	60%

Prostate (SU2C)	Metastatic Prostate Cancer, SU2C/PCF Dream Team (Robinson et al., Cell 2015)	90	60%
Cervical (TCGA)	Cervical Squamous Cell Carcinoma and Endocervical Adenocarcinoma (TCGA, Provisional)	175	59.30%
GBM (TCGA)	Glioblastoma Multiforme (TCGA, Provisional)	340	58.90%
GBM (TCGA 2013)	Glioblastoma (TCGA, Cell 2013)	324	57.50%
Liver (TCGA)	Liver Hepatocellular Carcinoma (TCGA, Provisional)	206	55.70%
GBM (TCGA 2008)	Glioblastoma (TCGA, Nature 2008)	99	48.10%
DLBC (TCGA)	Lymphoid Neoplasm Diffuse Large B-cell Lymphoma (TCGA, Provisional)	23	47.90%
Melanoma (TCGA)	Skin Cutaneous Melanoma (TCGA, Provisional)	167	45.60%
Pancreas (TCGA)	Pancreatic Adenocarcinoma (TCGA, Provisional)	83	45.10%
Bladder (MSKCC 2012)	Bladder Cancer (MSKCC, J Clin Oncol 2013)	43	44.30%
Prostate (TCGA)	Prostate Adenocarcinoma (TCGA, Provisional)	218	44.30%
Prostate (TCGA 2015)	Prostate Adenocarcinoma (TCGA, in preparation)	141	42.30%
Lung adeno (Broad)	Lung Adenocarcinoma (Broad, Cell 2012)	77	42.30%
Sarcoma (MSKCC)	Sarcoma (MSKCC/Broad, Nature Genetics 2010)	86	41.50%
Colorectal (TCGA)	Colorectal Adenocarcinoma (TCGA, Provisional)	237	38.50%
Uterine (TCGA)	Uterine Corpus Endometrial Carcinoma (TCGA, Provisional)	197	36.50%
Colorectal (TCGA pub)	Colorectal Adenocarcinoma (TCGA, Nature 2012)	85	33.10%
Uterine (TCGA pub)	Uterine Corpus Endometrioid Carcinoma (TCGA, Nature 2013)	115	31.70%
ACyC (MSKCC)	Adenoid Cystic Carcinoma (MSKCC, Nat Genet 2013)	19	31.70%
Prostate (Broad/Cornell 2013)	Prostate Adenocarcinoma (Broad/Cornell, Cell 2013)	15	26.80%
ACC (TCGA)	Adrenocortical Carcinoma (TCGA, Provisional)	23	25.60%
Breast (BCCRC Xenograft)	Breast cancer patient xenografts (British Columbia, Nature 2014)	29	25%
Prostate (MSKCC 2010)	Prostate Adenocarcinoma (MSKCC, Cancer Cell 2010)	38	19.60%
ccRCC (TCGA)	Kidney Renal Clear Cell Carcinoma (TCGA, Provisional)	102	19.30%
Prostate (Broad/Cornell 2012)	Prostate Adenocarcinoma (Broad/Cornell, Nature Genetics 2012)	18	16.50%

PCPG (TCGA)	Pheochromocytoma and Paraganglioma (TCGA, Provisional)	25	15.40%
pRCC (TCGA)	Kidney Renal Papillary Cell Carcinoma (TCGA, Provisional)	40	13.90%
Liver (AMC)	Liver Hepatocellular Carcinoma (AMC, Hepatology 2014)	28	12.10%
Bladder (MSKCC 2014)	Bladder Cancer (MSKCC, Eur Urol 2014)	13	11.90%
AML (TCGA pub)	Acute Myeloid Leukemia (TCGA, NEJM 2013)	22	11.50%
AML (TCGA)	Acute Myeloid Leukemia (TCGA, Provisional)	22	11.50%
ccRCC (TCGA pub)	Kidney Renal Clear Cell Carcinoma (TCGA, Nature 2013)	43	9.90%
Thyroid (TCGA)	Thyroid Carcinoma (TCGA, Provisional)	31	6.20%
chRCC (TCGA)	Kidney Chromophobe (TCGA, Cancer Cell 2014)	4	6.10%
chRCC (TCGA)	Kidney Chromophobe (TCGA, Provisional)	4	6.10%
Thyroid (TCGA pub)	Papillary Thyroid Carcinoma (TCGA, Cell 2014)	25	5%
Prostate (MSKCC 2014)	Prostate Adenocarcinoma CNA study (MSKCC, PNAS 2014)	4	3.80%
Glioma (TCGA)	Brain Lower Grade Glioma (TCGA, Provisional)	0	0%
